# Estimation of direct and indirect polygenic effects and gene–environment interactions using polygenic scores in case–parent trio studies

**DOI:** 10.1038/s41588-026-02601-2

**Published:** 2026-06-02

**Authors:** Ziqiao Wang, Luke Grosvenor, Debashree Ray, Tianyuan Cheng, Ingo Ruczinski, Terri H. Beaty, Heather Volk, Christine Ladd-Acosta, Nilanjan Chatterjee

**Affiliations:** 1 Department of Genome Sciences, University of Virginia, Charlottesville, VA USA; 2 Department of Biostatistics, Bloomberg School of Public Health, Johns Hopkins University, Baltimore, MD USA; 3 Division of Research, Kaiser Permanente Northern California, Pleasanton, CA USA; 4 Department of Mental Health, Bloomberg School of Public Health, Johns Hopkins University, Baltimore, MD USA; 5 Department of Epidemiology, Bloomberg School of Public Health, Johns Hopkins University, Baltimore, MD USA; 6 Department of Oncology, School of Medicine, Johns Hopkins University, Baltimore, MD USA

**Keywords:** Genetic association study, Autism spectrum disorders

## Abstract

We have proposed PGS-TRI, a framework for analyzing polygenic scores (PGSs) in case–parent trio studies that estimate the risk of an index condition associated with direct PGS effects, gene–environment interactions and asymmetrical maternal and paternal indirect effects. Simulations confirm its robustness in the presence of complex population structure and assortative mating. Applied to multi-ancestry autism spectrum disorders (ASD) trios (*n*_trio_ = 18,383), PGS-TRI yielded transmission-based direct effects of PGSs for ASD and other neurocognitive traits along a genetic ancestry continuum, and identified asymmetrical indirect effects of parental PGSs for body mass index and neurocognitive traits on children’s ASD risk. In a trio study of European and Asian orofacial clefts (OFCs) (*n*_trio_ = 1,904), PGS-TRI estimated direct and indirect effects of an established PGS and its interaction with maternal risk factors. Finally, we applied PGS-TRI to large-scale, transcriptome-wide and metabolome-wide traits to examine their direct and indirect effects on ASD and OFC risk.

## Abstract (web summary)

PGS-TRI, a framework designed for analyzing polygenic scores in case–parent trio studies, allows estimation of the risk of an index condition associated with direct and indirect polygenic effects and related gene–environment interactions.

## Main

Large genome-wide association studies (GWASs) of unrelated individuals have been widely used to derive polygenic scores (PGSs) for complex traits. Although it is standard practice to account for population stratification using genetic principal components (PCs), recent studies^1–5^ have shown that genetic effects can be overestimated due to residual confounding with geographical variations and assortative mating in the population. This complicates translational applications and interpretations for PGSs across various analyses, including risk predictions and Mendelian randomization analysis^6^. Family-based association studies^7^, which estimate genetic effects through within-family comparisons, can protect against such biases when assessing effects of individual genetic variants as well as PGSs. Furthermore, family-based studies with parental data can be used uniquely to estimate the indirect effects^8–11^ of parental genetic variation on offspring outcomes, possibly mediated through parental environmental factors, providing genetic-based evidence for the effect of parental exposures on children’s health outcomes.

Existing methods for estimating PGS effects associated with disease risks through family-based studies remain limited. One study^7^ separated the within-family and between-family effects of PGSs using random-effect models to account for family-specific effects. Another study^11^ pioneered the use of parental genotype data on probands to separate direct and indirect effects of PGSs on traits within families. These methods were developed for quantitative traits in randomly sampled families and are not suitable for other important study designs where families are ascertained through affected probands, such as case–parent trios, mother–child dyads or other family-based study designs. One recent study^12^ introduced the polygenic transmission disequilibrium test (pTDT) based on case–parent trio designs and detected evidence of polygenic risk for autism spectrum disorders (ASD), irrespective of the presence of high de novo variants in probands. The pTDT, however, does not provide estimates of effect sizes on a suitable risk scale, a critical gap for comparing risk estimates with population-based studies, estimating causal effects using Mendelian randomization studies or modeling interactions.

To address these limitations and meet the current needs for the analysis of PGSs in family-based studies, we introduced PGS-TRI. This method can be applied to case–parent trio study designs to estimate the risk of a condition in offspring associated with direct (inherited) effects of an index PGS and its interaction with environmental exposures (PGS × E) and indirect effects of parental PGS^8,10^. The method allows disease risk and PGS distribution to vary across families in a flexible manner, making it highly resilient to population stratification and assortative mating. We have shown that, under our modeling framework, the PGS distribution in ascertained families can be derived in a compact form and conveniently partitioned into transmission and parental components. Based on this factorization, we estimated the direct effect of the PGS and the effect of PGS × E interactions on offspring outcomes using the transmission component, while using a key scale parameter estimate from the parental PGS distribution. In addition we have shown how parental PGS data can be used to derive a simple and highly robust estimator for the difference in indirect effects of maternal and paternal PGS on an offspring’s outcome. We conducted extensive simulation studies to demonstrate the validity and power of PGS-TRI for detecting direct and indirect effects, and PGS × E interactions in the presence of complex population structures and assortative mating.

We applied PGS-TRI to a large multi-ancestry dataset of case–parent ASD trios ascertained from the Simons Powering Autism Research for Knowledge (SPARK) consortium^13^. We derived established PGSs for ASD, several neurocognitive traits and body mass index (BMI), and investigated the risk of ASD in the children associated with the direct and indirect effects of these scores. We observed direct effects for these scores of similar patterns and magnitude to those reported in population-based studies. Moreover, we observed significant indirect effects for the scores for BMI and several neurocognitive traits, but not for ASD itself. We further demonstrated that the heterogeneity in ASD–PGS effects across ancestry groups can be explained by a continuous attenuation of association strength, reflecting the genetic distance between the target population from the PGS training population. We further applied the proposed method to a trio study of orofacial clefts (OFCs)^14,15^, another developmental disorder known to be highly heritable. The analysis revealed a strong direct effect of an established PGS across European and Asian populations and multiple disease subtypes, but no evidence of any indirect effects. For both ASDs and OFCs, we further evaluated PGS × E interactions for several known maternal risk factors. Finally, we explored the effectiveness of PGS-TRI as a discovery tool by analyzing PGSs for gene-expression and metabolite traits, obtained from the OMICSPRED study^16^, to investigate their potential direct or indirect effects on the risk of the two conditions.

## Results

### Overview of methods

**Fig. 1:**
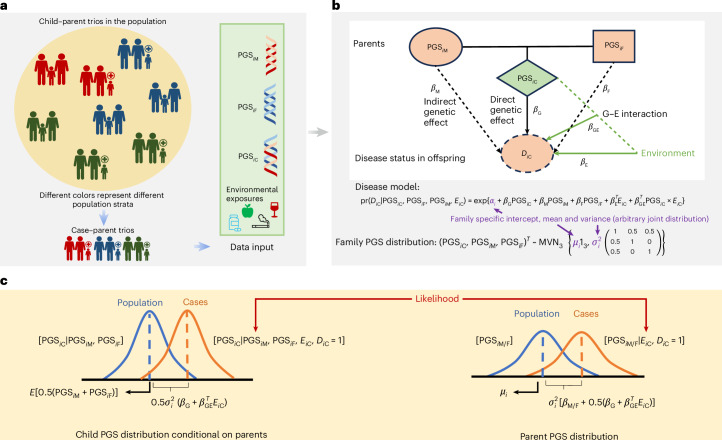
Figure illustrating our PGS-TRI model framework for case–parent trio family study designs. Population-level PGS direct genetic effect (*β*_G_), direct PGS–E interactions (**β**_GE_) and environmental effects (**β**_E_) associated with offspring’s outcome. Mother (*β*_M_) and father (*β*_F_) indirect genetic effects associated with the offspring’s outcome risk. PGS values of child (PGS_*i*C_), mother (PGS_*i*M_) and father (PGS_*i*F_) in family, *i* = 1, …, *N*. *D*_*i*C_, outcome status of the offspring in family *i* = 1, …, *N*. **a**, Study design and data input for PGS-TRI. **b**, Schematic diagram of direct, indirect genetic effects, and G × E interactions in the model setup of PGS-TRI. **c**, The distributions of child and parental PGS in case-parent trios exhibit mean shifts but unchanged variance relative to the general population. We partition the likelihood into transmission-based likelihood (child PGS distribution conditional on parents) and parental likelihood (parent PGS distribution).

An overview of the methods is presented in Fig. 1. Given genotype data from case–parent trios, we modeled the risk of the index condition on a log-linear scale in relation to direct PGS effects, PGS-by-environment interactions and indirect parental PGS effects. We showed that model parameter estimators can be derived in closed form. In particular, the direct effect of a PGS could be estimated using transmission disequilibrium statistics, appropriately scaled by estimates of within-family variance of the PGS. We showed that gene–environment interaction parameters can be estimated by covariance between transmission disequilibrium statistics and environmental factors across families, after suitable scaling. The difference in indirect effects of the parental PGSs can be estimated by the difference in PGS values between two parents, averaged across families, and suitably scaled by within-family variance parameters (Methods and Supplementary Note 1).

### Simulation studies

**Fig. 2:**
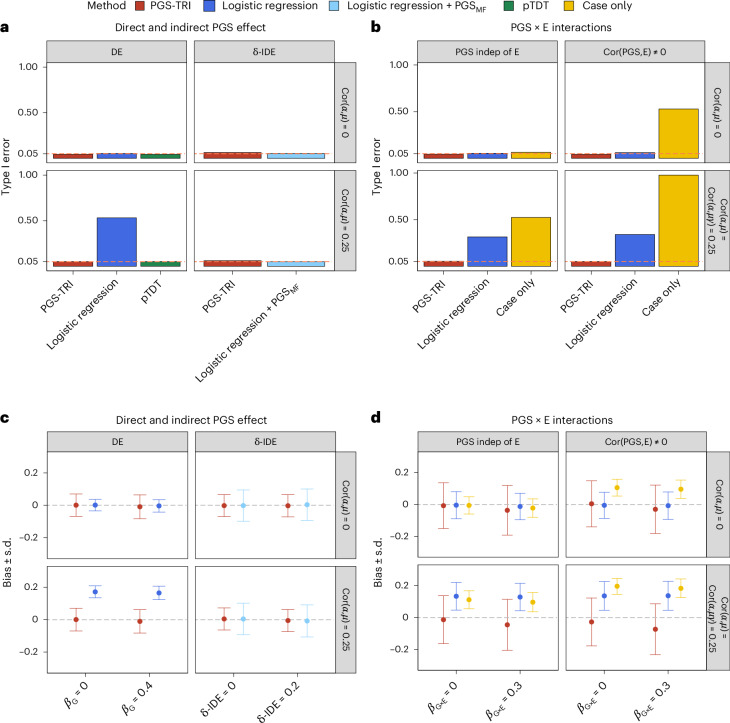
Performance of PGS-TRI and alternative methods for estimating parameters of PGS DEs, parental δ-IDEs and PGS × E interactions in simulation studies. **a**, Results shown for type I error of PGS DEs and δ-IDEs, when underlying true effects are 0. **b**, Type I error of PGS–E interaction, when underlying true main effect DE is 0.4. **c**, Data presented as bias ± empirical s.d. of the estimates for DEs and δ-IDEs, where bias = mean estimated value − true parameter value. **d**, Data presented as bias ± empirical s.d. of the estimates for the PGS–E interaction. The pTDT is implemented as an alternative method for testing DEs. Logistic regression is implemented for testing and estimation of DEs assuming that unrelated controls are available of the same size as the number of cases. Logistic regression is also implemented for testing and estimation of δ-IDEs, further assuming that parental genotypes are available for the unrelated cases and controls. Furthermore, a case-only method is also implemented for testing PGS × E interaction. Data are repeatedly simulated for 1,000 trios or 1,000 unrelated cases and 1,000 unrelated controls from the underlying population. Cor, correlation; indep, independent; logistic regression + PGS_MF_, logistic regression with child’s PGS, mother’s PGS and father’s PGS in the model.

**Extended Data Fig. 1:**
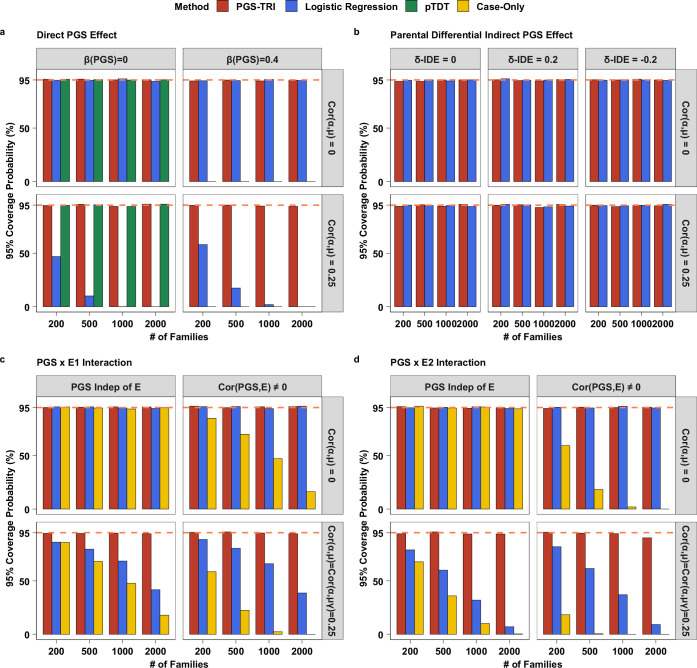
Coverage level of 95% confidence intervals of PGS-TRI and alternative methods for estimation of risk parameters in simulation studies. **a**, Coverage level for the estimation of DE. Logistic regression is implemented assuming unrelated controls are available of the same size as the number of cases. **b**, δ-IDE. Logistic regression is implemented further assuming that parental genotypes are available for the unrelated cases and controls. **c**, PGSxE1 interactions. **d**, PGSxE2 interactions. A case-only method is implemented for testing PGSxE interaction terms. Logistic regression is also implemented for testing of PGSxE assuming unrelated controls are available of the same size as the number of cases. For each type of parameter, results are shown in scenarios in the absence and the presence of population stratification bias. Data are repeatedly simulated for 1000 trios, or 1000 unrelated cases and 1000 unrelated controls from the underlying population.

**Extended Data Fig. 2:**
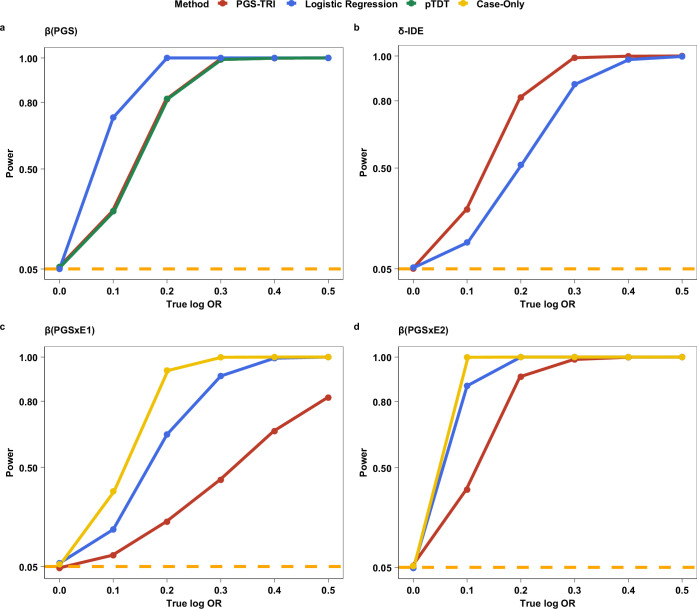
Power of PGS-TRI compared to alternative methods for the detection of risk parameters. **a**, DE. Here, pTDT is implemented as an alternative method for testing DE. Logistic regression is also implemented for testing of DE assuming unrelated controls are available of the same size as the number of cases. **b**, δ-IDE. Logistic regression is further implemented for the testing of δ-IDE assuming parental genotypes are available for the unrelated cases and controls. **c** and **d**, PGS by E interaction terms. The case-only method is implemented as an alternative method for testing PGSxE. Logistic regression is also implemented for testing of PGSxE assuming unrelated controls are available of the same size as the number of cases. For fair comparisons, power results are only presented in the absence of population stratification when logistic regression and the case-only method have no bias. Data are repeatedly simulated for 1000 trios, or 1000 unrelated cases and 1000 unrelated controls from the underlying population. All tests were two-sided and were conducted at a significance level of 0.05.

**Extended Data Fig. 3:**
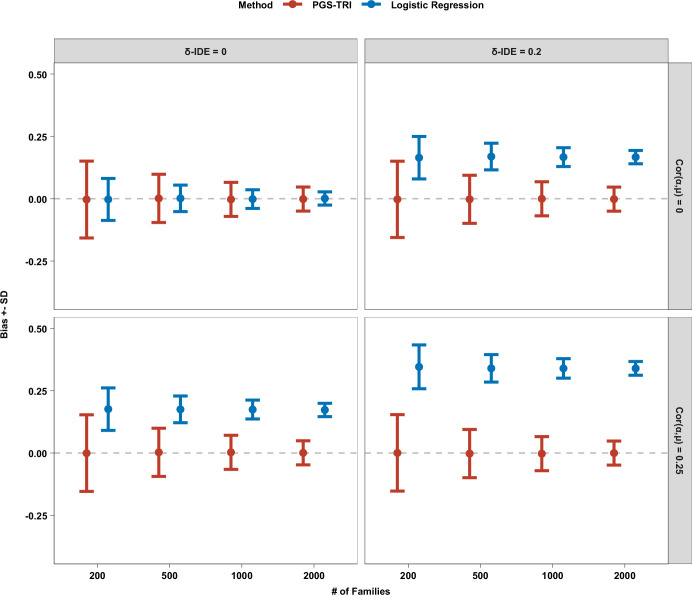
Biases and SD of estimates of DE of PGS using PGS-TRI and logistic regression when indirect effects of parental PGS are not incorporated into modeling. Data are presented as bias ± empirical standard deviation (SD) of the estimates for DE, where bias = mean estimated value − true parameter value. In the top left and bottom left panels, data were simulated assuming no $\delta$-IDE. In the top right and bottom right panels, data were simulated in the presence of $\delta$-IDE. Both PGS-TRI and logistic regression were fitted without the parental indirect effect parameters in the model. Data are repeatedly simulated for 200, 500,1000, and 2000 trios, and for case-control samples with 200, 500,1000, and 2000 unrelated cases and corresponding 200, 500,1000, and 2000 unrelated controls from the underlying population. All tests were two-sided and were conducted at a significance level of 0.05.

In simulation studies, where we directly generate data under the assumed model (Methods), we observed that PGS-TRI produces unbiased effect-size estimates, well-controlled type I error rates and calibrated confidence intervals (CIs) for all different types of parameters across a realistic range of population stratification scenarios (Fig. 2 and Extended Data Fig. 1). The pTDT, being a transmission-based method, also produces unbiased tests for direct genetic effects and has identical power to PGS-TRI across different scenarios. Standard logistic regression analysis of unrelated case–control participants produces biased inference for direct effects (DEs) in the presence of correlations between PGS mean and disease risks across families. Logistic regression and case-only analysis also produce significant bias for inference on gene–environment interaction parameters in the presence of complex population substructures across which disease risk, exposure distribution and PGS distribution co-vary. If parental genotype data were available for unrelated case–control participants, then a logistic regression model could also be used to estimate the magnitude of differential indirect effects (δ-IDEs). In the absence of population stratification, both methods are valid for the estimation of DEs and δ-IDEs, but logistic regression is more powerful for detecting DEs, whereas PGS-TRI is more powerful for detecting δ-IDEs (Extended Data Fig. 2). Furthermore, logistic regression can produce biased inference for DEs not only in the presence of population stratification but also in the presence of IDE when parental data are not available to account for such effects (Extended Data Fig. 3).

**Fig. 3:**
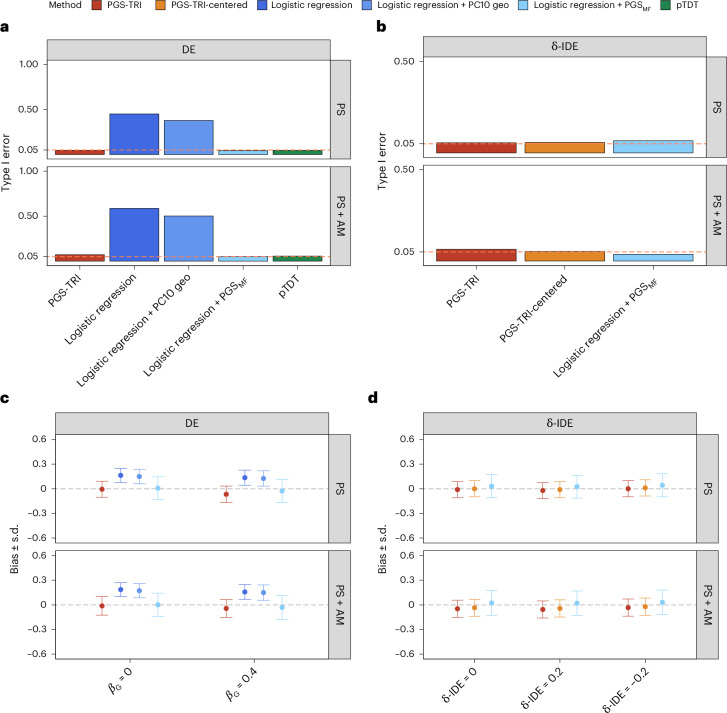
Performance of PGS-TRI and alternative methods for the estimation of genetic effects associated with EA PGS in the UKB-based simulation study. **a**, Results for type I error associated with the DE of the PGS. **b**, Type I error associated with the parental δ-IDE of the PGS. **c**, Data presented as bias ± empirical s.d. of the estimates for the DE, where bias = mean estimated value − true parameter value. **d**, Data presented as bias ± empirical s.d. of the estimates for the δ-IDE. PGS-TRI-centered subtracts the parental PGS difference in the UKB population from the numerator of the original PGS-TRI δ-IDE estimate. Among alternative methods, pTDT is implemented for testing of the DE. For testing of the DE, logistic regression is implemented for the analysis of unrelated cases and controls without any adjustment, or adjustment for the top ten genetic PCs constructed from the parental data, the assessment centers and the north and east birth coordinates of the parents, or logistic regression with parental PGSs in the model. For the testing and estimation of the δ-IDE using logistic regression, we assumed that parental genotype data are available on unrelated cases and controls. Data on children for matched pairs of UKB participants are repeatedly simulated and then a set of 1,000 case–parent trios or a set of 1,000 unrelated cases and 1,000 unrelated controls is further sampled for subsequent analysis. Parents were either matched by only geographical proximity to simulate effect of population stratification or geographical proximity and EA level to simulate the effect of both population stratification and assortative mating. AM, assortative mating; logistic regression + PC10 geo, logistic regression with the top ten genetic PCs, north and east birth coordinates and assessment centers as covariates in the model; PS, population stratification bias.

**Extended Data Fig. 4:**
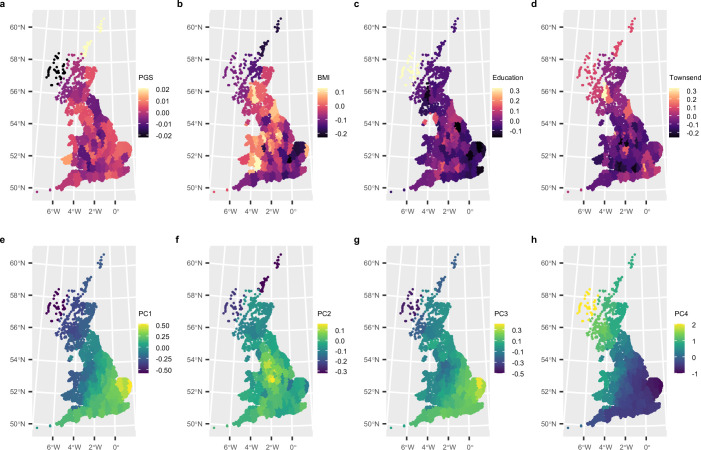
Geographical distributions of PGS and other environmental confounders of unrelated UK Biobank participants (considered as “parents” in our simulation study). Values of unrelated UK Biobank participants were plotted for **a**, Educational Attainment (EA) PGS. **b**, Body Mass Index (BMI). **c**, Educational Attainment (EA). **d**, Townsend Index, (**e**–**h**) Top 4 Principal Components. Individuals were grouped into 100 clusters based on their east and north co-ordinates of birthplaces using the K-means clustering. Colors represent the mean values of grouped individuals in each cluster on the map. The between-cluster correlation between BMI and EA-PGS is -0.59 (*P* = 1*10^−10^), the within-cluster correlation is -0.098 (*P* > 0.05). Parental BMI was treated as a hidden environmental confounding variable while simulating outcome status in children. All tests were two-sided and were conducted at a significance level of 0.05. UK Biobank individual coordinates were projected to the British National Grid coordinate reference system, defined as EPSG:27700 (OSGB36 / British National Grid), using R package sf.^73,74^.

**Extended Data Fig. 5:**
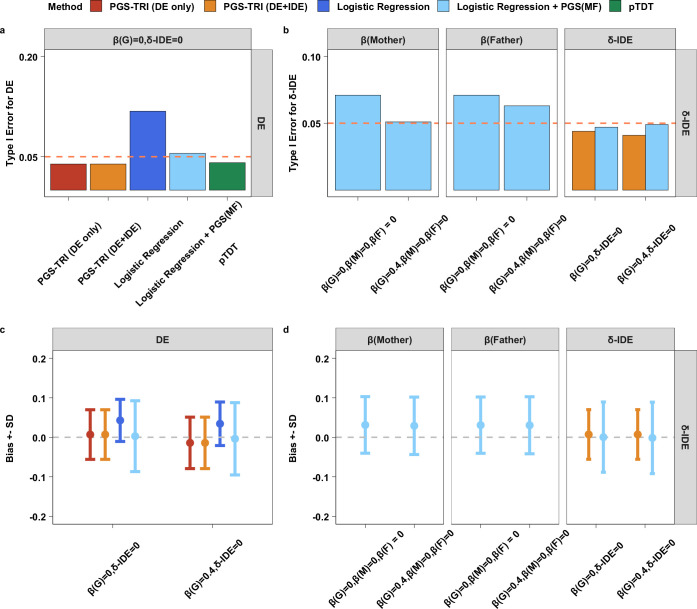
Simulation results under 20 generations of assortative mating effects using snipar comparing PGS-TRI, logistic regression, and pTDT. Type I error for the detection of (**a**) DE when $\delta$-IDE = 0, (**b**) $\delta$-IDE when DE = 0 or 0.4. (**c**) Data are presented as bias ± empirical standard deviation (SD) of the estimates for DE, where bias = mean estimated value − true parameter value. (**d**) Data are presented as bias ± empirical standard deviation (SD) of the estimates for parental indirect effects in logistic regression + PGS(MF) and $\delta$-IDE using PGS-TRI and logistic regression + PGS(MF). pTDT is implemented as an alternative method for testing DE. Logistic regression is also implemented for testing of DE assuming unrelated controls are available of the same size as the number of cases. All tests were two-sided and were conducted at a significance level of 0.05. Data are repeatedly simulated for 1000 trios, or 1000 unrelated cases and 1000 unrelated controls from the underlying population. All tests were two-sided and were conducted at a significance level of 0.05. **PGS-TRI (DE only)**: PGS-TRI assuming direct effect only; **PGS-TRI (DE** + **IDE)**: PGS-TRI assuming there are both direct and indirect parental effects in the model; **Logistic Regression**: logistic regression with children’s PGS in the model; **Logistic Regression** + **PGS(MF)**: logistic regression with children’s PGS, mothers’ PGS and fathers’ PGS in the model.

We further confirmed the robustness of PGS-TRI to realistic patterns of population substructure and assortative mating by considering analyses of an established educational attainment (EA) PGS in the UK Biobank (UKB) study. In this setting, we first matched unrelated male and female participants to form ‘parents’, and then we simulated children’s genotype data under Mendel’s law of inheritance. We observed that, when participants are matched by geographical proximity, there is significant across-family correlation (*P* = 1.03 × 10^−10^) between EA PGS and parental BMI, which is treated as a hidden ‘environmental’ confounder for the simulations of disease risk in children (Extended Data Fig. 4a–d). In this setting, logistic regression analysis of unrelated cases and controls, even after adjustment for genetic PCs and geographical coordinates, can produce significantly biased inference for DEs (Fig. 3). When we further added EA to the matching criterion, that is, allowing for assortative mating in addition to geographical population structure, there was increased bias in logistic regression. We observed that PGS-TRI generally produces both unbiased tests and effect-size estimates in all scenarios. We observed a slight difference between maternal and paternal mean PGS values in the overall UKB sample (as further illustrated in Fig. 3b,d) and this led to a slightly inflated type I error and estimation bias for δ-IDEs using PGS-TRI, but these errors were controlled when we centered δ-IDEs using underlying population difference in the PGS mean between the sexes. Similarly, for the simulation of 20 generations of assortative mating using snipar^17^, PGS-TRI remains unbiased for testing and estimation of both PGS DEs and δ-IDEs (Extended Data Fig. 5). Another observation is that, although the logistic model with parental PGS adjustment can largely account for assortative mating effects, its estimates of DEs and δ-IDEs are less efficient than those from PGS-TRI (Extended Data Fig. 5c,d).

### Polygenic risk for ASD

**Fig. 4:**
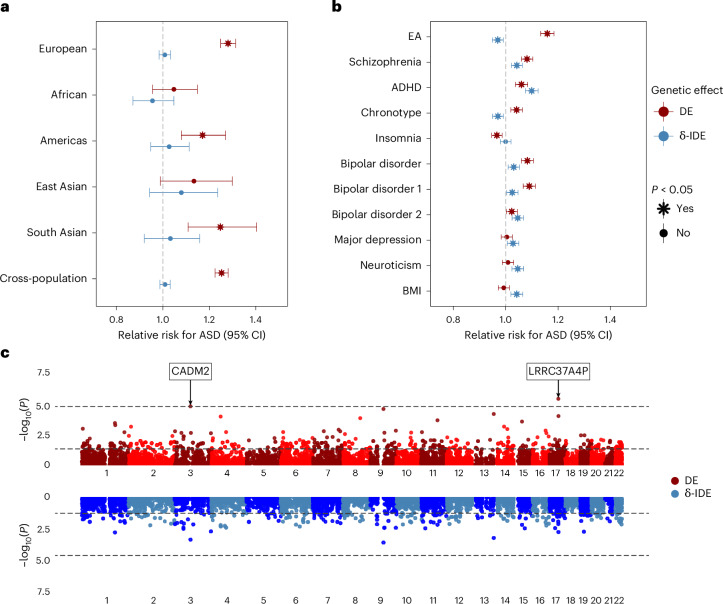
SPARK study results for DEs and δ-IDEs of ASD. **a**, Data presented as the RR estimate and 95% CI for DEs and δ-IDEs of ASD–PGS (PGS ID: PGS000327) on ASD risk across multiple ancestry groups. **b**, Data presented as the RR estimate and 95% CI for DEs and δ-IDEs of PGSs for multiple neurocognitive traits on ASD risk (only shown for combined population analysis due to sample size in ancestral subpopulations). The *P* values shown in the figure were not adjusted for multiple comparisons. After Bonferroni’s correction, six traits for DEs and five traits for δ-IDEs remained statistically significant in the cross-population analysis (see detailed results including exact *P* values in Supplementary Table 5). In **a** and **b**, PGSs are standardized by subtracting PC projections based on the 1000 Genomes Project and Human Genome Diversity Project (1,000G + HGDP) reference data of independent individuals across populations and divided by the population s.d. calculated using the same reference data so that RR corresponds to an increase in risk per s.d. unit increase in PGS value. **c**, Miami plot showing results from the transcriptome-wide association study using PGS-TRI of the risk of ASD associated with the DEs and δ-IDEs of PGSs for gene-expression traits available from the OMICSPRED study in homogeneous EUR families (*N*_trio_ = 12,813). After multiple hypothesis testing adjustment, CADM2 (*P* = 1.96 × 10^−5^, FDR = 0.048) and LRRC37A4P (*P* = 4.92 × 10^−6^, FDR = 0.024) were identified. Detailed results are given in Supplementary Table 6. All tests were two sided and conducted at a significance level of 0.05. BMI was used as a negative control for the DE.

We first examined the association of the ASD–PGS derived from the European ancestry (EUR) iPSYCH study^18^ with ASD risk across different ancestry groups (Fig. 4a and Supplementary Tables 1 and 2). We observed, for the EUR population (*n*_trio_ = 12,813), that PGS-TRI produced a transmission-based estimate of DE (relative risk (RR) = 1.28, 95% CI = [1.25, 1.31]) slightly smaller than the reported estimate (odds ratio (OR) = 1.33, 95% CI = [1.30, 1.36]) from the iPSYCH study^18^, which predominantly used unrelated EUR case–control samples. Thus, our estimate of effect size suggests no evidence of significant bias in prior population-based GWASs due to unadjusted population stratification. We also detected evidence of a significant direct effect of the PGS on ASD risk within American (*n*_trio_ = 1,302) and south Asian (*n*_trio_ = 554) families, with the corresponding effect-size estimates being of similar magnitude to those derived from EUR families. However, we did not find significant evidence of direct effects of the PGS on ASD risk in African (*n*_trio_ = 792) or east Asian (*n*_trio_ = 415) families. We did not detect any evidence of nonzero δ-IDE associated with the ASD–PGS on the risk of ASD.

**Fig. 5:**
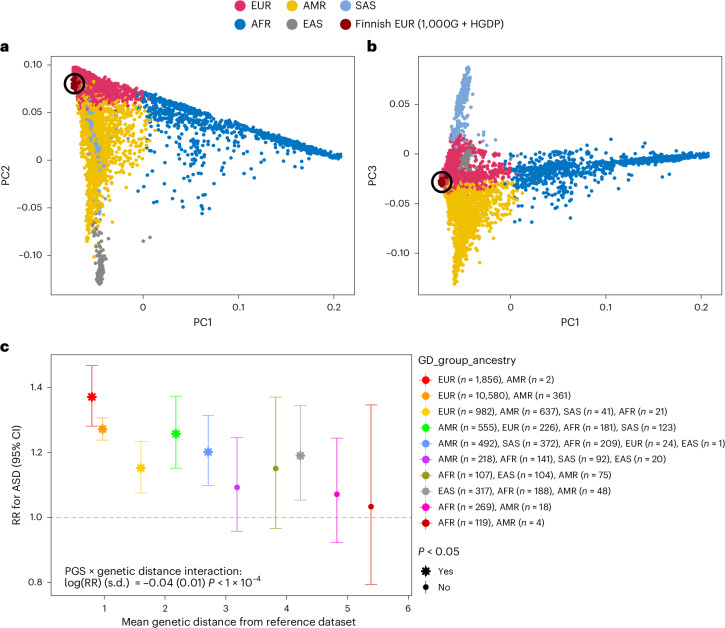
SPARK study results for PGS × context interactions of ASD. **a**, PC analysis of genetic data in children with ASD in SPARK. PC1 vs PC2. PCs were projected based on eigenvectors generated using genetics data in 1000G + HGDP-unrelated individuals. Dark-red points are Finnish individuals from 1000G + HGDP. **b**, PC1 vs PC3. **c**, Data presented as the RR estimate and 95% CI for DEs for ASD risk using ASD–PGS (PGS ID: PGS000327) in ten groups of equal genetic distance intervals using PGS-TRI. This demonstrates PGS × context interactions, using PGS-TRI (*P* = 3.8 × 10^−5^). Detailed results are given in Supplementary Table 3. Each group consists of families of different ancestry groups (in total *N*_trio_ = 18,383). Each ancestry group in the legend notation is based on the offspring’s ancestry; note that parents may be from a different ancestry group. All tests were two sided and conducted at a significance level of 0.05. Genetic distance is calculated as the Euclidean distance of the top five PCs between each ASD child and the center of the unrelated Finnish individuals from the 1000G + HGDP reference data. The PGS is standardized by subtracting PC projections based on 1000G + HGDP reference data of independent individuals across populations and divided by the group-specific population s.d. calculated using the same reference data, so that RR corresponds to an increase in risk per s.d. unit increase in PGS value. AFR, African; AMR, admixed American; EAS, east Asian; SAS, south Asian.

Based on the observed pattern of heterogeneity of effect sizes of ASD–PGS across ancestry groups, we hypothesized that the DE of this PGS on ASD risk decreases as the genetic distance between the PGS training population (EUR) and testing sample increases. To validate this, we analyzed data from all families (*n*_trio_ = 18,383), including those with parents from different ancestries (*n*_trio_ = 5,570), and examined how the DE of the ASD–PGS vary by genetic distance between the target sample and the training population (Fig. 5). The analysis revealed that the log-risk of autism associated with the DE of the PGS decreases linearly with increasing genetic distance (Fig. 5c). This linear relationship is further supported by the highly significant PGS × context interaction term (*P* < 1 × 10^−4^). These results remain robust irrespective of whether genetic distances were defined by the top two, five or ten PCs (Supplementary Table 3).

We explored gene–environment interactions of the ASD–PGS and several specific prenatal and perinatal environmental factors on ASD risk (Supplementary Table 4). We did not observe any strong evidence of interactions, indicating polygenic risk and environmental factors generally act multiplicatively on the risk of ASD. We observed nominal evidence that maternal alcohol consumption during pregnancy modified the DE of the ASD–PGS on offspring risk in all families and EUR families (*P* = 0.043 and 0.032, during pregnancy compared to never drinkers).

We next examined the DEs and IDEs of PGSs for several neurocognitive traits and BMI on ASD risk (Supplementary Table 5). The results revealed significant DEs for most of these scores, with patterns like those observed in population-based studies (Fig. 4b). As expected, we did not observe any DE of BMI–PGS on autism risk, because children’s genetic predisposition to BMI is not anticipated to influence their own risk for developmental conditions like ASD. Accordingly, BMI serves as a negative control in this analysis. It is interesting that the analysis identified maternally mediated IDEs (δ-IDE > 0) of PGSs for many of these traits, with five remaining significant after Bonferroni’s adjustment for multiple testing. The corresponding effect sizes suggest that δ-IDEs are larger than the DEs for several traits, including attention-deficit hyperactivity disorder (ADHD), bipolar disorder 2, major depression and neuroticism. We also observed significant evidence of maternally mediated effects of BMI–PGS on ASD risk in children (*P* < 2 × 10^−4^), aligning with epidemiological findings on the influence of maternal obesity on child health outcomes. In an additional sensitivity analysis of EUR ancestry families, centering the asymmetrical IDE estimates based on the female–male differences observed among married UKB participants of EUR ancestry, we observed δ-IDEs mostly remained highly significant, with even stronger associations for BMI and chronotype (Supplementary Table 5).

**Extended Data Fig. 6:**
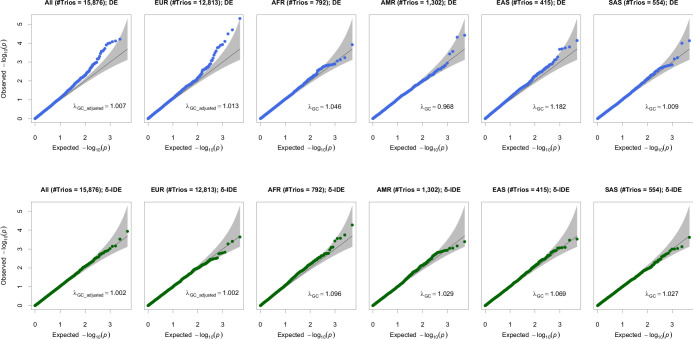
Quantile-quantile plot (QQ plot) of p-values generated by PGS-TRI for the transcriptome-wide association study of autism risk in the SPARK study. The DE and $\delta$-IDE of PGS associated with a total of 4,907 transcriptomic traits are tested. The diagonal lines correspond to expected p-values percentiles under the null hypothesis and the shaded regions represent 95% confidence bands based on beta distribution order statistics. **DE**: PGS direct effect; ${\boldsymbol{\delta }}$-**IDE**: differential parental indirect genetic effect; ${{\boldsymbol{\lambda }}}_{{\boldsymbol{GC}}\_{\bf{adjusted}}}$: scaled genomic inflation factor to a study with 1000 subjects; ${{\boldsymbol{\lambda }}}_{{\boldsymbol{GC}}}$: genomic inflation factor, a value that equals 1 represents no inflation. All tests were two-sided and were conducted at a significance level of 0.05.

Finally, in the analysis of OMICSPRED-generated PGSs for gene-expression and metabolite traits using PGS-TRI, we observed that genetically predicted *CADM2* expression showed significant DE on ASD risk in both cross-ancestry and EUR-only analyses (Fig. 4c and Supplementary Table 6). *CADM2* is predominantly expressed in the brain and known to play a crucial role in synapse organization and neuronal activity. GWASs have linked common variants near *CADM2* as associated with a wide variety of psychiatric and neurobehavioral traits^19–21^, EA^22^, obesity^23,24^ and latent factors underlying insulin resistance and psychiatric traits^25^. We further validated the results using the precomputed genetic weights^26^ from Genotype-Tissue Expression (GTEx) v8 in brain tissues and found nominal significance of genetically predicted *CADM2* expression in the substantia nigra (Supplementary Table 7) on ASD risk. We did not detect evidence (false discovery rate (FDR) <0.05) of any indirect effect of either gene-expression or metabolite scores (Supplementary Tables 8–10). The quantile–quantile plots for both DEs and δ-IDEs associated with 4,907 gene-expression scores show that PGS-TRI controls type I error rates well in transcriptome-wide analyses (Extended Data Fig. 6), suggesting its potential as a well calibrated discovery tool for large-scale, genetically predicted molecular studies using case–parent trio designs.

### Polygenic risk of OFCs

**Fig. 6:**
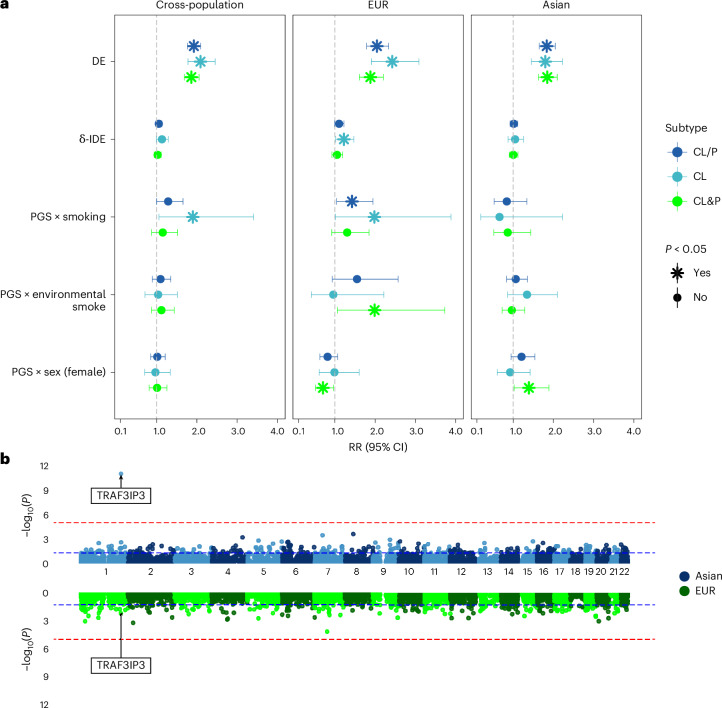
Results from the analysis in the GENEVA study of OFC trios. **a**, Data presented as the RR estimate and 95% CI for different OFC subtypes associated with the DEs and δ-IDEs of the OFC–PGS (PGS ID PGS002266) and the interaction of the OFC–PGS with several maternally medicated risk factors. The PGS was standardized within each ancestry group by population mean and s.d. calculated using 1000G reference data of independent individuals, so that the RR corresponded to an increase in risk per s.d. unit increase in PGS value. The *P* values shown in the figure were not adjusted for multiple comparisons. **b**, Miami plot showing results from the transcriptome-wide association study using PGS-TRI of the risk of the combined OFC CL/P (Asian *N*_trio_ = 891, EUR *N*_trio_ = 575) associated with the DEs of PGSs for gene-expression traits available from the OMICSPRED study. After multiple hypothesis testing adjustment, TRAF3IP3 was identified in Asians (*P* = 1 × 10^−11^, FDR = 5 × 10^−8^). Detailed results are given in Supplementary Tables 15 and 16. All tests were two sided and conducted at a significance level of 0.05.

We applied PGS-TRI to analyze the risk of OFCs using EUR and Asian ancestry trios available from the GENEVA study (Fig. 6 and Supplementary Tables 11–14). We first examined the risk of various OFC subtypes with a PGS incorporating 24 SNPs defined by an earlier study (PGS catalog ID: PGS002266)^27,28^. We found highly significant and consistent levels of DEs of this PGS on the risk of OFCs across different subtypes, including cleft lip without cleft palate (CL alone), cleft lip with cleft palate (CL&P) and cleft lip with or without cleft palate (CL/P, CL alone and CL&P combined), and across both populations. The strength of these associations are of similar magnitude to those reported in previous studies using both population-based and family-based samples^28^. In our analysis, we did not find any evidence of an association between this PGS and the cleft palate (CP alone) subtype, an anatomically and embryologically distinct subtype. This was not surprising, considering that the original PGS was developed based on studies of CL/P subtypes only. In the cross-ancestry population (combined EUR and Asian populations) analysis, we did not observe evidence of δ-IDE of the parental PGS on the risk of OFCs in offspring. In the EUR-only analysis, however, we observed nominal-level evidence of maternally mediated δ-IDE of the PGS on the risk of CL alone (*P* = 0.03).

We further used PGS-TRI to explore the interaction of the DE of OFC–PGS with several known prenatal environmental risk factors and offspring’s sex on the OFC subtypes (Fig. 6a). In the EUR population, we detected evidence of interaction of the PGS with maternal smoking during pregnancy on the risk of CL alone and combined CL/P and, to a lesser extent, with maternal environmental smoking exposure in CL&P. These interactions were not significant in Asians, but the power for detecting interaction in this population was very low because only a small proportion (3%) of the affected probands were exposed to maternal smoking (Supplementary Table 12). We further observed evidence of PGS by sex interactions in EUR on CL&P risk, but an interaction effect in the opposite direction for the Asian population, which likely canceled each other in cross-ancestry population analysis.

**Extended Data Fig. 7:**
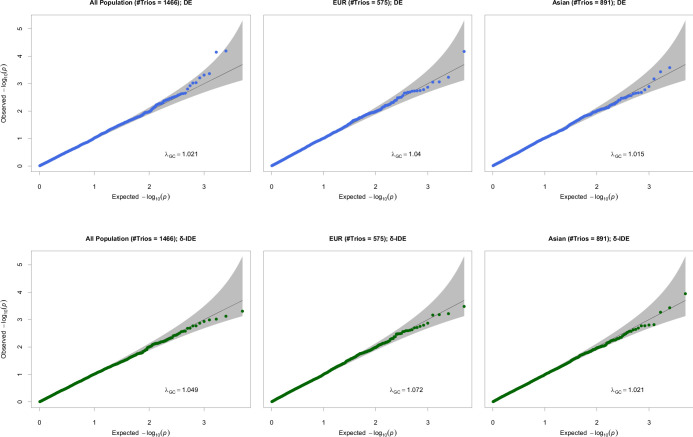
Quantile-quantile plot (QQ plot) of p-values generated by PGS-TRI for the transcriptome-wide association study of the risk of OFC CL/P subtype in the GENEVA study. The DE and $\delta$-IDE of PGS associated with a total of 4,991 transcriptomic traits are tested. The diagonal lines correspond to expected p-values percentiles under the null hypothesis and the shaded regions represent 95% confidence bands based on beta distribution order statistics. **DE**: PGS direct effect; ${\boldsymbol{\delta }}$**-IDE**: differential parental indirect genetic effect; ${{\boldsymbol{\lambda }}}_{{\boldsymbol{GC}}}$: genomic inflation factor, a value that equals 1 represents no inflation. All tests were two-sided and were conducted at a significance level of 0.05.

Finally, we examined the risk of OFCs (CL/P and combined OFCs) associated with DEs and δ-IDEs of transcriptomic PGSs generated by the OMICSPRED study^29^. We detected strong evidence of DE of genetically predicted expression of *TRAF3IP3* on the risk of CL/P (cross-ancestry *P* = 2.0 × 10^−12^) with the strength of association appearing to be stronger in the Asian population compared with the EUR population (heterogeneity test *P* = 0.01; Fig. 6b and Supplementary Table 15). The genetic score of *TRAF3IP3* expression involved 44 SNPs within the *cis* region and had a proportion of variance explained *R*^2^ = 0.139 in the EUR population. An intronic SNP rs2235370 of *TRAF3IP3* has been previously reported as a sentinel variant associated with the risk of CL/P in a previous GWAS cross-ancestry meta-analysis^30^. The DE of *TRAF3IP3-*PGS on CL/P risk became insignificant when we removed eight SNPs in linkage disequilibrium with rs2235370 (Pearson’s *r*^2^ > 0.3), but the DE of the genetic score based only on the eight SNPs remained highly significant (cross-ancestry *P* = 6.5 × 10^−13^). Application of genotypic TDT^31^ further showed a strong association of each of the individual eight SNPs with the risk of CL/P (Supplementary Table 16). These results combined suggested that the presence of a haplotype in this region has a protective effect on OFC risk, likely mediated by the expression level of the gene *TRAF3IP3*. We did not detect any evidence of δ-IDEs of the transcriptomic PGSs on OFC risk (Extended Data Fig. 7 and Supplementary Table 17). We also did not find evidence of DEs or δ-IDEs of metabolomic PGSs on OFC risk (Supplementary Tables 18 and 19).

## Discussion

We developed a new analytical framework, PGS-TRI, for the analysis of PGSs in case–parent trio studies. It enabled transmission-based estimation of the risk of the outcome in offspring, accounting for the direct effects of inherited PGSs and their interaction with environmental factors. Furthermore, the method leveraged observed asymmetry in PGS values between parents within families to estimate maternally or paternally mediated IDEs on the offspring outcomes. We conducted simulation studies across various realistic scenarios, including one involving an EA PGS in the UKB study, to demonstrate the robustness of the proposed method against population structure and assortative mating. Our applications of PGS-TRI to two distinct developmental conditions, ASD and OFCs, provided transmission-based estimates of effect sizes for established PGSs and addressed concerns over biases in previous studies caused by uncorrected population structure, assortative mating or indirect genetic effects. We also applied PGS-TRI to new analyses exploring polygenic gene–environment interactions in these two conditions. Finally, we used PGSs for gene-expression and metabolite traits to examine any evidence of their DEs and IDEs on these two conditions.

Case–parent trio studies and related analytical methods have a significant history and long-standing utility in the field of genetic epidemiology, especially for developmental health conditions in children. Originally, the TDT^32^ was proposed as an allelic test for linkage and association and it was believed that it would form the basis of future GWASs^33^. Several other key studies noted that transmission-based testing and risk estimation can be conducted based on marker genotype data without having to assume multiplicative effects of underlying alleles^34,35^. Subsequently, a series of methods was proposed for the transmission-based analysis of multi-allelic markers^36^, complex pedigrees^37^, gene–environment interactions^38–40^, indirect (also referred to in prior studies as ‘nurturing’)^41,42^ and imprinting or parent-of-origin effects^40,42^, and a more general class of distribution-free methods for family-based association testing also emerged^43–45^. As GWASs required very large sample sizes for detecting small polygenic effects, studies based on unrelated cases and controls became widely popular due to the ease of recruiting. In the post-GWAS era, however, there has now been renewed interest in the use of case–parent trios and other family-based studies for more robust characterizations of risks associated with GWAS-identified genetic effects^7,11,12^. In addition, there are practical considerations that make it more feasible to collect data from parents in families with young affected children than collecting large samples of unrelated cases and controls. Thus, a family trio design is particularly well suited for modern and increasingly common developmental outcomes such as ASD and OFCs, among others.

The transmission-based method for PGS analysis in case–parent trios was proposed based on the deviation of observed PGS values of children from its expected value under Mendel’s law of transmission, which is represented by the mid-parental PGS values^12^. Although the method provided a valid test, the underlying statistics do not provide valid effect estimates. Here we demonstrate that unbiased risk estimation requires scaling of transmission disequilibrium statistics by estimates of within-family variance of the PGS, which we now incorporate into the PGS-TRI method. Furthermore, our method, which is motivated by the likelihood of the trio data, allows for the modeling of PGS–environment interactions within the transmission-based framework and can incorporate IDEs of parental PGSs on children’s outcomes. Within our framework, an estimate of the asymmetrical maternal and paternal IDEs of PGSs can be obtained from the average difference in PGS values between parents across families, scaled by the average within-family variance. Focusing on this difference—rather than on maternal and paternal effects separately—yields a parameter that is both biologically meaningful and more robust to the effects of population stratification and assortative mating. We used data from the SPARK consortium to obtain transmission-based estimates of ASD risk associated with predefined PGS of ASD. Our results validated previously reported risks in EUR ancestry populations for ASD–PGS. There is also a critical need to assess the portability of ASD–PGSs in non-EUR ancestries. Our analysis of the interaction between an established ASD–PGS and the genetic ancestry continuum shows that the transmission-based estimate of effect size for the PGS diminishes as an individual’s ancestry deviates further from the training population. Although such a continuous relationship between PGS effects and genetic ancestry has been shown in population-based studies^46^, we have shown this phenomenon through family-based studies. We observed that such PGS by context interactions have important implications for transferability across diverse ancestry populations, but they cannot be interpreted mechanistically because PGS predictive effects may vary due to differences in allele frequency and linkage disequilibrium patterns of underlying SNPs. Our results further show that the PGS and the subset of prenatal and perinatal environmental risk factors examined in this study have multiplicative effects on the risk of ASD.

We further observed significant DEs of PGSs for several other neurocognitive traits, including EA, schizophrenia, ADHD, chronotype, insomnia and bipolar disorders. We also observed evidence of potential δ-IDEs of parental PGSs for many of these same traits on children’s risk of ASD. However, our simulation studies indicate that asymmetrical distributions of PGSs between mothers and fathers in trio designs can also result from differential family participation driven by parental traits, such as maternal educational status. Therefore, we cannot exclude the possibility that some δ-IDEs reflect parental gender-specific selection bias in the SPARK families related to these traits (Supplementary Figs. 1–3). Furthermore, we identified gender asymmetry in PGS distribution among UKB participants, suggesting potential selection bias in general population cohorts by these traits. However, the magnitude of asymmetry in the UKB was substantially lower than observed among SPARK parents and, for some traits, the direction of asymmetry was even reversed between the two studies (Supplementary Table 20).

We also applied our new method to a new investigation of the polygenic risk of OFCs using data from the GENEVA study^14^. This analysis established transmission-based risk estimates for a predefined PGS across different OFC subtypes in both EUR and Asian ancestry groups. We identified modest evidence of nonmultiplicative interaction of the DE of an OFC–PGS and maternal smoking, as well as environmental maternal tobacco smoke exposure during pregnancy. Prior genome-wide SNP–environment interaction studies^47^, including but not limited to GENEVA samples, have not revealed genome-wide significant findings. Joint tests for genetic associations and gene–environment interactions had indicated modest evidence of gene-by-maternal smoking interactions for rs7541797 near PAX7, but little evidence was found for gene-by-environmental tobacco smoking^47^. In our PGS-based analysis, on the other hand, we found some evidence of aggregated SNPs by smoking interaction effects across different known loci. In the future, large and diverse studies including experimental model systems are needed to further characterize gene–environment interactions in the etiology of OFCs.

Overall, our studies of gene–environment interactions across OFCs and ASD and power analysis (Supplementary Figs. 4–7) suggest that PGSs and maternal risk factors generally show multiplicative effects on the relative-risk (RR) scale for both of the developmental conditions. When genes and environment act multiplicatively on the RR scale, it is expected that lifestyle interventions will have the largest impact on absolute risk of diseases for individuals who have the highest genetic susceptibility^48–50^. In the context of developmental traits, the implications of such gene–environment interaction for parental counseling and targeted intervention need further investigation.

Despite its strengths, our framework has several limitations. It estimates only the difference in IDEs between parents, not each parent’s effect separately; this could be addressed by incorporating stronger parametric assumptions. We modeled gene–environment interactions only with respect to the DE of a PGS and further research is merited to extend the model to allow the possibility of interactions of environmental factors with indirect parental effects. Our assumption of gene–environment independence within ‘homogeneous’ families is highly robust to the presence of population structure and assortative mating. However, there could be also direct correlation between a PGS and environmental exposures due to pleiotropic effects. As discussed before, estimates of δ-IDEs can be biased in the presence of differential selection bias associated with parental sex or when children do not live with both parents and, consequently, are not exposed to the corresponding parental IDEs. Finally, our molecular trait PGS analysis using OMICSPRED may lack power for developmental outcomes, because these scores were trained on adult samples and their predictive utility in children depends on the stability of molecular quantitative trait loci across life stages^51^.

The proposed framework for conducting PGS analysis in case–parent trios opens numerous avenues for further research, including applying analogous analyses in other types of ascertained families such as mother–child dyads, case–parent trios with unaffected siblings and more complex pedigrees that may be ascertained through multiple probands. In addition, there is the potential to extend this model for joint analysis of multiple, possibly correlated PGSs, which could be useful for applications such as multivariable Mendelian randomization analysis^52^. Case–parent trio designs are widely used for many developmental and early childhood conditions due to the feasibility of collecting parental data and biosamples. Moreover, for many late-onset diseases, studies have collected genetic data on extended families ascertained with specific conditions like breast cancer for understanding risks associated with rare high-penetrant mutations. Our proposed method, along with its future extensions, can facilitate PGS analysis in ascertained families, enabling robust characterization of associated risks, both by a PGS itself and together with rare mutations and nongenetic risk factors.

## Methods

### Ethics section

This work used datasets from the UKB^53^, the SPARK consortium^13^, Research Match: Genes and Environment Autism Research Study (GEARS) study and the GENEVA study^14,15^. The UKB (https://www.ukbiobank.ac.uk) is under application no. 17731 and was approved by the North West Multi-centre Research Ethics Committee under reference no. 21/NW/0157. All participants provided written informed consent to participate. The SPARK and the GEARS studies used extant genomic and newly collected prenatal environmental data, using the GEARS environmental questionnaire, from participants enrolled in the SPARK study. The SPARK-integrated whole-exome sequencing (iWES) v3 dataset was generated from the SPARK study, led by researchers at Boston Children’s Hospital (study reviewed and approved by the institutional review board (IRB) of WIRB Copernicus Group). The GEARS study was reviewed and approved by the Johns Hopkins Bloomberg School of Public Health (protocol nos. IRB00007694 and IRB00021723) IRB. Written informed consent was obtained from all participants included in the analysis. These data are owned and distributed by the Simons Foundation Autism Research Initiative (SFARI). Aligning with the Common Rule definition of human research, as of March 2025, SFARI does not require IRB approval for requests of de-identified dataset biospecimens, including the SPARK iWES v3 and GEARS datasets. The GENEVA study was reviewed and approved by the IRB of each participating site, including the Johns Hopkins Bloomberg School of Public Health (protocol no. RPN 91-06-10-03-2).

### Modeling direct genetic effects and gene–environment interactions

We assumed that PGSs for the index condition and other traits of interest can be evaluated for family trio participants, using published meta-data from prior association studies. Our goal is to investigate the association of these PGSs with an index condition, such as ASD, using case–parent trios. In a trio, let PGS_C_, PGS_M_ and PGS_F_ denote the PGS values of the child, mother and father respectively. In addition, let *D*_C_ denote the binary outcome status of the child. We assumed that *i* = 1, …, *N* families have been sampled following the case–parent trio design. We also assumed that a set of environmental exposures, denoted by **E**_*i*C_, has been ascertained for each child across the different families. We assumed that prospective disease risk of the child in the population follows a log-linear model:

1 $$\begin{array}{l}{\mathrm{pr}}({D}_{i{\rm{C}}}{|{\mathrm{PGS}}}_{i{\rm{C}}},{{\mathrm{PGS}}_{i{\rm{F}}},{\mathrm{PGS}}_{i{\rm{M}}},{\bf E}_{i{\rm{C}}}})\begin{array}{cl}= & {\mathrm{pr}}({D}_{iC}{|{\mathrm{PGS}}}_{i{\rm{C}}},{{\bf {E}}_{i{\rm{C}}}})=\end{array}\exp \left\{\,{\alpha }_{i}+{\beta }_{{\rm{G}}}{\mathrm{PGS}}_{i{\rm{C}}}\right.\\ \,\,\,\,\,\,\,\,\,\,\,\,\,\,\,\,\,\,\,\,\,\,\,\,\,\,\,\,\,\,\,\,\,\,\,\,\,\,\,\,\,\,\,\,\,\,\,\,\,\,\,\,\,\,\,\,\,\,\,\,\,\,\,\,\,\,\,\,\,\,\,\,\,\left.+{{\mathbf{\upbeta}}_{E}^{{\bf{T}}}}{{\bf {E}}_{i{\rm{C}}}}+{{\mathbf{\upbeta}}_{\mathrm{GE}}^{{\bf{T}}}}{\mathrm{PGS}}_{i{\rm{C}}}\times {{\bf {E}}_{i{\rm{C}}}}\right\}.\end{array}$$

Here pr is probability, ${\beta }_{{\rm{G}}}$ is the direct genetic effect (DE), **β**_GE_ is the vector of PGS × **E** interaction effects, **β**_E_ is the vector of main environment effects and **T** refers to a vector transpose. In equation (1), we assumed no indirect genetic effects, that is, within each family the parental PGS values affect only the child’s disease risk mediated through the child’s own PGS value, but we relaxed this assumption later (see subsequent sections and Supplementary Note 1). In addition, this model incorporates family-specific intercept terms, *α*_*i*_, without any further assumption about their distribution, thereby allowing disease risks to vary arbitrarily across families. We used the log-linear model because it yielded closed-form estimators and allowed for the direct estimation of RR parameters, which are more interpretable in many epidemiological contexts. Although logistic regression is more commonly used—primarily because it constrains predicted probabilities between 0 and 1—it estimates ORs, which may be less intuitive. For rare diseases such as the developmental conditions studied here, the RR and OR tend to converge, making the two models nearly equivalent in practice. Although logistic regression is standard in case–control designs, prior studies^38,54^ have shown that the log-linear- model is more suitable for case–parent and case-only designs, and rare disease assumptions are commonly invoked for comparing estimates with those from the logistic model.

We assumed that the joint distributions of PGS across families in the underlying population follow trivariate normal distributions of the form:

2 $${\left({\mathrm{PGS}}_{i{\rm{C}}},{\mathrm{PGS}}_{i{\rm{M}}},{\mathrm{PGS}}_{i{\rm{F}}}\right)}^{T} \sim {\mathrm{MVN}}_{3}\left\{{\mu }_{i}{1}_{3},{\sigma }_{i}^{2}\left(\begin{array}{ccc}1 & 0.5 & 0.5\\ 0.5 & 1 & 0\\ 0.5 & 0 & 1\end{array}\right)\right\}.\,$$

Here the correlation of 0.5 between PGS values of individual parents and children followed from Mendel’s law of inheritance. We allowed family-specific mean (*μ*_*i*_) and variance (${\sigma }_{i}^{2}$) terms without imposing any assumptions about their distributions. We assumed that, within a family, PGS for the parents were independently distributed.

We have explained here how a model of equation (2) can flexibly account for both population structure and assortative mating. We conceptualized a sampling mechanism where, for each family sampled, we assumed that it belongs to a unique subpopulation of ‘homogeneous’ families with distinct genetic ancestry and trait characteristics influencing assortative mating. The number of these subpopulations can be arbitrarily large, making each subpopulation at an extremely fine level. It is assumed that random mating occurs within these homogeneous fine-level subpopulations, implying that the PGS correlation between partners is zero within these subpopulations, but not necessarily across them. We observed that the framework has similarity with classic models for assortative mating^55^ which also assume random mating in a generation conditional on trait characteristics. Finally, we assumed ${{\bf {E}}_{i{\rm{C}}}} \perp ({{\rm{PG}}{\rm{S}}}_{i{\rm{C}}},{{\rm{PG}}{\rm{S}}}_{i{\rm{M}}},{{\rm{PG}}{\rm{S}}}_{i{\rm{F}}})$, but allow the distribution **E**_*i*C_ to remain unspecified. From a population perspective, this assumption can again be viewed as gene–environment independence within highly homogeneous subpopulations, but the model can still accommodate gene–environment correlation at the population level which may arise due to population substructure and assortative mating.

### Retrospective likelihood and parameter estimation

The retrospective likelihood for the case–parent trio data in each family *i* can be decomposed into an offspring’s (*L*_*i*C_) and a parent’s (*L*_*i*P_) component as:

$$\begin{array}{lll}{L}_{i} & = & {\rm{pr}}({{\rm{PG}}{\rm{S}}}_{i{\rm{C}}},{{\rm{PG}}{\rm{S}}}_{i{\rm{M}}},{{\rm{PG}}{\rm{S}}}_{i{\rm{F}}}|{{\bf {E}}_{i{\rm{C}}}},{D}_{i{\rm{C}}}=1)\\ & = & {\rm{pr}}({{\rm{PG}}{\rm{S}}}_{i{\rm{C}}}{|{\rm{PG}}{\rm{S}}}_{i{\rm{M}}},{{\rm{PG}}{\rm{S}}}_{i{\rm{F}}},{{\bf {E}}_{i{\rm{C}}}},{D}_{i{\rm{C}}}=1)\times {\rm{pr}}({{\rm{PG}}{\rm{S}}}_{i{\rm{M}}},{{\rm{PG}}{\rm{S}}}_{i{\rm{F}}}|{{\bf {E}}_{i{\rm{C}}}},{D}_{i{\rm{C}}}=1)\\ & \mathop{=}\limits^{{\scriptscriptstyle {\rm{def}}}} & {L}_{i{\rm{C}}}\times {L}_{i{\rm{P}}}.\end{array}$$

Under the above model, the likelihood components associated with children ${(L}_{i{\rm{C}}})$ and parental PGS $({L}_{i{\rm{P}}})$ data for each family can be derived in terms of the following normal distributions (see Supplementary Note Section 1 for detailed derivation):

$$\begin{array}{l}\left[{\mathrm{PGS}}_{i{\rm{C}}}|{{{\mathrm{PGS}}_{i{\rm{M}}},{\mathrm{PGS}}_{i{\rm{F}}},{\bf E}_{i{\rm{C}}}},D}_{i{\rm{C}}}=1\right] \sim N({\mu }_{i{\rm{C}}}+0.5{\sigma }_{i}^{2}({\beta }_{\rm{G}}+{{\mathbf{\upbeta}}_{\rm{GE}}^{\bf{T}}}{{\bf{E}}_{i{\rm{C}}}}),0.5{\sigma }_{i}^{2}),\end{array}$$

and

$$\left[{\mathrm{PGS}}_{i{\rm{M}}/i{\rm{F}}}|{{{\bf{E}}_{i{\rm{C}}}},D}_{i{\rm{C}}}=1\right] \sim N\left({\mu }_{i}+0.5{\sigma }_{i}^{2}\left({\beta }_{\rm{G}}+{{\mathbf{\upbeta}}_{\rm{GE}}^{{\bf{T}}}{\bf{E}}_{i{\rm{C}}}}\right),{\sigma }_{i}^{2}\right).$$

Maximum-likelihood estimation based on $L=\displaystyle {\prod }_{i=1}^{N}{L}_{i}$ can be complex due to the presence of large dimensional nuisance parameters ${({\mu }_{i},{\sigma }_{i})}_{i=1}^{N}$. Instead, we proposed a combination of likelihood-based and moment-based estimation. First, we observed that the likelihood ${L}_{{\rm{C}}}=\displaystyle {\prod }_{i=1}^{N}{L}_{i{\rm{C}}}$ is informative for estimating ${\beta }_{{\rm{G}}}$ and $\bf{\upbeta }_{\rm{GE}}$, but one complication is that it requires estimates of family-specific variance parameters ${\sigma }_{i}^{2}$. We then observed that, under the above model, the PGS values for two parents within each family are expected to have identical distribution and, thus, ${\hat{\sigma }}_{i}^{2}=0.5{({{\rm{PG}}{\rm{S}}}_{i{\rm{M}}}-{{\rm{PG}}{\rm{S}}}_{i{\rm{F}}})}^{2}$ provides an unbiased estimator for ${\sigma }_{i}^{2}$, *i* = 1, ‥. *N*. We can then obtain estimates of ${\beta }_{{\rm{G}}}$ and $\bf{\upbeta }_{\rm{GE}}$ based on the likelihood *L*_C_ with plugged-in values for ${\hat{\sigma }}_{i}^{2}$. Under the above framework, we have shown that the final estimator can be derived in an analytical form as a solution to a weighted least-square problem as:

3 $${\hat{\bf{\beta}}}={({\bf{E}}^{\rm{T}}{\hat{\bf{W}}}{\bf{E}})}^{-1}{\bf{E}}^{\rm{T}}{\bf{Z}},$$

where $${\upbeta}={({\upbeta}_{\rm{G}},{{\mathbf{\upbeta}}_{\rm{GE}}^{\rm{T}}})}^{{\rm{T}}}$$, $${\bf{E}}=({\bf{E}}_{1}^{\rm{T}},\cdots ,{\bf{E}}_{n}^{\rm{T}})^{\rm{T}}$$, $${\bf{E}}_{i}=(1,{\bf{E}}_{i{\rm{C}}}^{{\rm{T}}})^{{\rm{T}}}$$, $$\hat{\bf{W}}={\rm{diag}}({\hat{\sigma }}_{1}^{2},\cdots ,{\hat{\sigma }}_{n}^{2})$$, $${\mathbf{Z}}=2({{\rm{PG}}{\rm{S}}}_{1{\rm{C}}}-{\mu }_{1{\rm{C}}},\cdots ,{{{\rm{PG}}{\rm{S}}}_{{\rm{NC}}}-\mu }_{{\rm{NC}}})^{{\rm{T}}}$$ and $${\mu }_{i{\rm{C}}}=0.5({{\rm{PG}}{\rm{S}}}_{i{\rm{M}}}+{{\rm{PG}}{\rm{S}}}_{i{\rm{F}}})$$.

In the special case when no gene–environment interaction terms are incorporated, the estimate of the DE of a PGS takes the simple form:

4 $${\hat{\beta }}_{{\rm{G}}}=\frac{2\displaystyle {\sum }_{i=1}^{N}({\mathrm{PG}{\rm{S}}}_{i{\rm{C}}}-0.5({\mathrm{PG}{\rm{S}}}_{i{\rm{M}}}+{\mathrm{PG}{\rm{S}}}_{i{\rm{F}}}))/N}{\displaystyle {\sum }_{i=1}^{N}{\hat{\sigma }}_{i}^{2}/N}.$$

It is noteworthy that the numerator of equation (4) forms the basis of the pTDT test^12^. In pTDT, the numerator is normalized by the variance of average PGS values of the parents across families. However, our derivation of equation (4) suggests that obtaining an unbiased estimate of effect size for the PGS in TDT-type analysis requires normalization of the transmission disequilibrium statistics, that is, the numerator, by an estimate of within-family variance. Intuitively, in all ascertained designs, such as case–control and case–parent trio studies, association tests effectively compare PGS values between cases and suitable controls or pseudo-controls. The observed mean difference in PGS reflects both the true effect size ($\beta$) and the variance of the PGS in the sampled population. As PGS variance differs between unrelated and related individuals—especially in the presence of population structure—even a fixed effect size can yield contrasts of different magnitudes across designs. Therefore, to obtain a consistent and interpretable estimate of $\beta$, it is necessary to standardize the contrast using an appropriate variance term specific to the design.

We also note that the general form of equation (3), which incorporates gene–environment interactions, has an intuitive interpretation. The term ${\bf{E}}^{{\rm{T}}}{\bf{Z}}$ essentially represents the sample covariance between the transmission disequilibrium statistic and the environmental covariates across families. Under the null of no gene–environment interaction, transmission within a family is expected to be independent of family-level or parent-specific environmental factors (for example, maternal diet). Our derivation shows that departures from this null expectation in case–parent trio data provide evidence of gene–environment interaction on the multiplicative scale. However, we note that such transmission-based tests may not be appropriate when the environmental factor reflects a child’s own trait value, because these values can remain correlated with the child’s PGS even after conditioning on parental PGSs.

### Incorporating indirect parental genetic effects

Next, we extend equation (1) to incorporate indirect parental genetic effects as:

5 $$\begin{array}{l}\begin{array}{l}\begin{array}{l}\mathrm{pr}({D}_{i{\rm{C}}}|\mathrm{PG}{{\rm{S}}}_{i{\rm{C}}},{\mathrm{PG}{{\rm{S}}}_{i{\rm{F}}},\mathrm{PG}{{\rm{S}}}_{i{\rm{M}}},{\bf E}_{i{\rm{C}}}})\\ =\exp \{{\alpha }_{i}+{\beta }_{{\rm{G}}}\mathrm{PG}{{\rm{S}}}_{i{\rm{C}}}+{\beta }_{{\rm{M}}}\mathrm{PG}{{\rm{S}}}_{i{\rm{M}}}+{\beta }_{{\rm{F}}}\mathrm{PG}{{\rm{S}}}_{i{\rm{F}}}+{{\mathbf{\upbeta}}_{\bf{E}}^{{\rm{T}}}{\bf{E}}_{i{\rm{C}}}}+{{\mathbf{\upbeta} }_{\mathrm{GE}}^{{\rm{T}}}}\mathrm{PG}{{\rm{S}}}_{i{\rm{C}}}\times {{\bf {E}}_{i{\rm{C}}}}\},\end{array}\end{array}\end{array}$$

where ${\beta }_{{\rm{M}}},{\beta }_{{\rm{F}}}$ capture indirect effects of parental PGS on the disease risk of the children not mediated through the children’s genotypes.

The likelihood components associated with children (*L*_*i*C_) remain unchanged after incorporating the indirect genetic effects. We show, however, that the conditional distribution of PGS values in the mother or father in the *i*th ascertained family, when parental effects are incorporated, needs to be updated as $${L}_{i{\rm{P}}}=[{\mathrm{PGS}}_{i{\rm{M}}/i{\rm{F}}}|{{E}_{i{\rm{C}}},D}_{i{\rm{C}}}=1]$$$$\sim N\left({\mu}_{i}+{\sigma}_{i}^{2}\left[{\beta}_{{\rm{M}}/{\rm{F}}}+0.5({\beta}_{\rm{G}}+{\mathbf{\upbeta}}_{\mathrm{GE}}^{\bf{T}}{\bf{E}}_{{i}{\rm{C}}})\right],{\sigma}_{i}^{2}\right)$$. Now we observed that, unlike the previous setting, here the two parents within a family could have asymmetrical distribution depending on the difference in the magnitude of their IDEs ($${\beta }_{{\rm{M}}}$$ and $${\beta }_{{\rm{F}}}$$). We can exploit this parental asymmetry in PGS distribution to derive an estimator for the difference of parental indirect genetic effects (δ-IDE) as:

6 $$\hat{\delta }-\mathrm{IDE}={\hat{\beta }}_{{\rm{M}}}-{\hat{\beta }}_{{\rm{F}}}=\frac{{\sum }_{i=1}^{N}({\rm{PG}}{{\rm{S}}}_{i{\rm{M}}}-{\rm{PG}}{{\rm{S}}}_{i{\rm{F}}})/N}{{\sigma }_{{\rm{sum}}}^{2}/N}.$$

We further showed, in the presence of indirect effects, an approximately unbiased estimator for ${\sigma }_{\mathrm{sum}}^{2}/N=\displaystyle {\sum }_{i=1}^{N}{\sigma }_{i}^{2}/N$ can be derived as:

$${\hat{\sigma }}_{\mathrm{sum}}^{2}/N=\mathop{\sum }\limits_{i=1}^{N}{\hat{\sigma }}_{i}^{2}/N\approx \frac{1}{2(N-1)}{\mathop{\sum }\limits_{i=1}^{N}\left[({\mathrm{PGS}}_{i{\rm{M}}}-{\mathrm{PGS}}_{i{\rm{F}}})-\mathop{\sum }\limits_{i=1}^{N}({\mathrm{PGS}}_{i{\rm{M}}}-\mathrm{PG}{S}_{i{\rm{F}}})/N\right]}^{2}.$$

Furthermore, when the indirect effects of parental PGSs is incorporated, we observed that the form of the estimates of direct effect parameters **β** as shown in equation (3) remains unchanged, but the estimates ${\hat{\sigma }}_{i}^{2}$, *i* = 1, .‥ *N* in defining the weight matrix $\hat{\bf{W}}={\rm{diag}}({\hat{\sigma }}_{1}^{2},\cdots ,{\hat{\sigma }}_{N}^{2})$ needs to be modified as ${\hat{\sigma }}_{i}^{2}=0.5[(\mathrm{PGS}_{i{\mathrm{M}}}-{\mathrm{PGS}}_{i{\mathrm{F}}})-\displaystyle {\sum }_{i=1}^{N}({\mathrm{PGS}}_{i{\mathrm{M}}}-{\mathrm{PGS}}_{i{\mathrm{F}}})/N]^{2}$ to obtain more accurate estimator of the quantity ${{\bf {E}}^{\rm{T}}}{\bf{WE}}$.

Equation (6) provides an intuitive test for asymmetrical indirect parental effects. In ancestrally homogeneous populations, allele frequencies are generally expected to be equal across sexes, so the expected difference in PGS between mothers and fathers is 0. Assortative mating induces within-pair covariance but does not alter this expectation. Thus, any nonzero mean difference in case–parent trios can signal asymmetrical IDE of parental PGS on offspring risk. However, if population-level mean PGS values differ by sex in the parental population, for example, due to asymmetrical selection, then we expect $E({\rm{PG}}{{\rm{S}}}_{i{\rm{M}}}-{\rm{PG}}{{\rm{S}}}_{i{\rm{F}}}|{D}_{i{\rm{C}}}=1)={(\mu }_{{\rm{M}}}-{\mu }_{{\rm{F}}})+{\sigma }_{i}^{2}{\delta }_{{\rm{MF}}}$, where ${\mu }_{{\rm{M}}}$ and ${\mu }_{{\rm{F}}}$ denote the PGS mean for males and females in the underlying population. Thus, if sex-specific allele frequency for certain traits is suspected for the parental generation, we may further use an external dataset to obtain the difference in PGS between women and men to center our estimate of the indirect effect as:

$${\hat{\delta}}-{\mathrm{IDE}}-{\mathrm{centered}}=\frac{{\sum}_{i=1}^{N}({\rm{PG}}{\rm{S}}_{i{\rm{M}}}-{\rm{PG}}{\rm{S}}_{i{\rm{F}}})/N-({\mu}_{{\rm{M}}}-{\mu}_{{\rm{F}}})}{{\sigma}_{\rm{sum}}^{2}/N}.$$

In our autism application, we used data from the UKB to obtain external estimates of male–female differences in PGS values for various secondary traits and used them to carry out sensitivity analyses for the detected δ-IDEs.

Detailed derivations of all mathematical results and asymptotic variance estimators are presented in Supplementary Note 1.

### Simulation studies

We conducted three types of simulation studies to evaluate the performance of the proposed method under complex population substructure and assortative mating. In the first setting, we simulated PGS values in >1 million randomly sampled trios from trivariate normal distribution based on equation (2) and disease status in children under the full model equation (5), and then further selected families with affected children (*D* = 1). We varied ${\rho }_{{\rm{G}}}={\rm{cor}}({\alpha }_{i},{\mu }_{i})$ to create different scenarios of population-stratification bias, with a value of 0 indicating no relationship between variation in disease risk and PGS distribution across the underlying substructure—a scenario where we do not expect any bias in population-based association studies. For the investigation of gene–environment interactions, we incorporated a binary (${E}_{1}$) and a continuous (${E}_{2}$) variable in the disease model. We allowed complex interrelationships among baseline disease risk $({\alpha }_{i})$, PGS mean (${\mu }_{i}$) and exposure means (${\gamma }_{i1}$ and ${\gamma }_{i2}$) across families, in manners known to affect the estimation of gene–environment interactions using unrelated individuals^56^.

We designed the second simulation setting to investigate the robustness of different methods for estimation of simulated effects of EA–PGS, a score that is known to be highly confounded with environmental factors due to population substructure^57,58^ in the UKB study (www.ukbiobank.ac.uk). We built EA–PGSs using 4,483 independent SNPs (*r*^2^ < 0.05 within 250 kb) and weights reported in previous work (PGS Catalog ID: PGS002440)^59^ for the UKB participants. To simulate geographical population structure, we matched unrelated UKB males and females within assessment centers (UKB field ID 54) and by birth locations defined by north and east coordinates (UKB field IDs 129 and 130). In addition, we matched pairs based on both geographical proximity and similarity in EA (UKB field ID 6138) to simulate the additional effect of assortative mating. For each matched pair of UKB participants, we simulated children by generating their individual genotype values based on Mendel’s law of transmission. We further simulated children’s disease status based on equation (5), where a ‘hidden’ environmental variable, defined as the average of BMI values (UKB field ID 21001) of the two parents, was introduced to influence disease risk.

In the third setting, we used an existing tool snipar^17^ to simulate genotype data for 100,000 families across 1,000 independent SNPs, incorporating 20 generations of assortative mating with the parental phenotype correlation at 0.5, and a continuous phenotype influenced by direct genetic effects and assortative mating. To simulate children’s disease status, we used equation (5), including children’s PGS and parental indirect genetic effects, as well as mid-parental phenotype residuals regressing out parental PGS values as a family-level covariate to create an assortative mating effect in children’s disease outcome.

We also conducted additional simulation studies to evaluate potential effects of selection bias on parameter estimates associated with DEs and IDEs obtained from PGS-TRI (Supplementary Figs. 1–3). Details on all simulation settings, including the matching algorithm used for the UKB simulation, can be found in Supplementary Note 2.

### Data analysis for application of PGS-TRI to ASD

We next demonstrated the application of PGS-TRI by analyzing genotype and epidemiological data available on *n*_trio_ = 18,383 case–parent trios from the SPARK study^13^ (https://sparkforautism.org/) (Supplementary Table 1). The key objectives included: (1) obtaining transmission-based estimates of the DE of the most advanced ASD–PGS to date built from prior studies, and comparing such an effect size from that reported from prior case–control studies; (2) assessing the portability of European-derived PGSs to non-European populations across ancestry groups and continuum of genetic ancestry; (3) evaluating the association of PGSs for multiple neurocognitive traits with ASD risk using the proposed transmission-based method; (4) characterizing the nature of interaction of ASD–PGS and prenatal exposure on ASD risk; and (5) discovering potential new associations of ASD risk with DEs and δ-IDEs of PGSs for gene-expression and obesity-related metabolite traits. We allowed the ancestry group of each family to be determined by the ancestry group of their offspring (Supplementary Table 1). Families where parents and the offspring share the same genetic ancestry group are defined as homogeneous families (*N*_homo_ = 15,876). For most of the analyses presented here, we used homogeneous families; however, for evaluating portability of PGS across the genetic ancestry continuum, we included all 18,383 trios.

Specifically, we examined ASD risk associated with DEs and IDEs of pre-constructed PGSs for ASD^18^ and 11 other cognitive and mental health-related traits that have been previously studied for potential links to ASDs^12,16,60–62^, including EA^63^, schizophrenia^64^, strictly defined lifetime major depressive disorder^65^, bipolar disorder^66^, bipolar disorder I^66^, bipolar disorder II^66^, neuroticism^63^, insomnia^63^, chronotype^63^, BMI^63^ and ADHD^67^ across different ancestry groups. The PGS for ASD itself were defined based on the largest GWAS to date conducted by the iPSYCH consortium and involved a total of 26,637 SNPs. We standardized all the PGSs across the continuum of genetic ancestry by mean adjustment^68^ of the raw PGSs against the top five genetic PCs, using the 1000 Genomes Project and Human Genome Diversity Project (1kGP + HGDP)^69^ as a reference dataset and then divided the PGSs residuals by ancestry-specific standard errors obtained from the reference dataset. This allowed interpretation of underlying risk parameters, that is, RRs, in a standard unit scale across ancestries.

We further examined ASD–PGS by context interactions, defined by the genetic distance on the continuous scale among all populations. Here genetic distance^46^ is the Euclidean distance of the top 5 genetic PCs between each ASD child and the center of 98 unrelated Finnish individuals from 1kGP + HGDP to represent the PGS training population (north European) from the iPSYCH consortium. Using the homogeneous families, we further examined gene–environment interactions with several prenatal environmental factors, information being collected in SPARK participants using questionnaires designed by the GEARS study. Finally, we examined the association of ASD risk with genetically predicted levels of gene expression and metabolites using thousands of genetic scores generated by the OMICSPRED project^29^. Here the underlying hypothesis is that genetically predicted biomarker levels, as captured by the underlying PGSs, could affect ASD risk through DEs or IDEs. We did note a caveat that, because the PGSs for biomarkers have been derived based on adult samples, the DEs of PGSs on children’s outcome are possible only if the same PGSs also predict biomarker levels in the fetal state and/or early childhood. Anticipating limited power for this analysis, we included only those biomolecular traits predicted with accuracy, *R*^2^ ≥ 0.1, by the underlying genetic scores according to the internal validation in OMICSPRED and those included at least 5 SNPs in the underlying model. This criterion resulted in the evaluation of a total of 4,907 gene-expression levels and 27 highly correlated obesity-related metabolites. Additional details of data preprocessing and covariate coding can be found in Supplementary Note 3.

### Data analysis for application of PGS-TRI to OFCs

We also applied PGS-TRI to investigate the polygenic risk of nonsyndromic OFCs using case–parent trio data from the GENEVA study^14,15^. This analysis included a total of 778 European and 1,126 east Asian ancestry trios (see Supplementary Tables 11 and 12 for the distribution of trios by subtypes and exposure). We used these trio data to examine the effects of a predefined OFC–PGS on the risk of OFCs across different subtypes and ancestry groups, and its interaction with prenatal exposure to maternal smoking, maternal alcohol consumption, use of multivitamins during pregnancy and prenatal environmental tobacco smoke exposure^47,70^. PGS for cleft lip with or without cleft palate (CL/P) were constructed using 24 SNPs and their respective weights sourced from the PGS Catalog^27,28^. We standardized the OFC–PGS by ancestry-specific standard errors obtained from the 1000 Genomes Phase 3 Project^69^. Finally, we also examined the risk of OFCs (CL/P) associated with the DEs and δ-IDEs of genetically predicted gene expressions and metabolite levels using genetic scores generated from the OMICSPRED project^29^. Additional details of data preprocessing and covariate coding can be found in Supplementary Note 3.

### Statistics and reproducibility

Our proposed method, PGS-TRI, was applied to estimate direct and asymmetrical indirect genetic effects and to assess gene–environment interactions (including ASD–PGS by context interactions) for all PGSs and genetically predicted transcriptome-wide and metabolome-wide traits within the SPARK consortium and GENEVA study. All hypothesis tests were two sided and based on Wald’s test statistics. Data analysis code to reproduce the results is available in our GitHub repository at https://github.com/ziqiaow/PGS.TRI and https://github.com/ziqiaow/PGS-TRI-Analysis and via Zenodo at 10.5281/zenodo.19189683 (ref. ^71^) and 10.5281/zenodo.19353771 (ref. ^72^).

### Reporting summary

Further information on research design is available in the Nature Portfolio Reporting Summary linked to this article.

## Online content

Any methods, additional references, Nature Portfolio reporting summaries, source data, extended data, supplementary information, acknowledgements, peer review information; details of author contributions and competing interests; and statements of data and code availability are available at 10.1038/s41588-026-02601-2.

## Supplementary information

Supplementary Information Supplementary Figs. 1–6, Notes (including statistical derivations, details of simulation study and data applications).

Reporting Summary

Peer Review File

Supplementary Tables Supplementary Tables 1–20.

## Data Availability

Summary statistics of the results of all PGS-TRI and pTDT analyses in the SPARK consortium and GENEVA study, irrespective of significance level, are available in Supplementary Tables 1–20. The GWAS summary statistics that we used to calculate PGSs were downloaded from the PGS Catalog^27^. For individual-level genetic and phenotypic data, GENEVA datasets are available in dbGaP through the accession no. phs000094.v1.p1. For individual-level phenotypical and genetic data on the SFARI Base, approved researchers can obtain the SPARK population dataset described in this study by applying at https://base.sfari.org. The SFARI Base accession ID is ‘SFARI_DS631850’. GRCh37 and GRCh38 reference genome data from the Phase 3 1000 Genome Project are available from https://www.internationalgenome.org/data. PC-based ancestry information and CRCh38 reference genome data (the gnomAD v3.1.2) from 1000 Genome + HGDP Project are available from https://gnomad.broadinstitute.org/downloads#v3. The detailed population information was extracted from the International Genome Sample Resource: https://www.internationalgenome.org/data-portal/sample. Access to UKB individual-level data can be requested from https://www.ukbiobank.ac.uk/enable-your-research/apply-for-access. The GTEx v8 genetic scores for gene expression of brain tissues have been downloaded from http://gusevlab.org/projects/fusion/#gtex-v8-multi-tissue-expression. OMICSPRED genetic scores have been downloaded from https://www.omicspred.org/.

## Code availability

The software PGS-TRI and the pTDT are publicly available via GitHub at https://github.com/ziqiaow/PGS.TRI and via Zenodo at 10.5281/zenodo.19189683 (ref. ^71^). A website hosting the tutorials is available at https://ziqiaow.github.io/PGS.TRI/. The data analysis and simulation code in this manuscript are available via GitHub at https://github.com/ziqiaow/PGS-TRI-Analysis and via Zenodo at 10.5281/zenodo.19353771 (ref. ^72^).

## References

[1] Kerminen, S. et al. Geographic variation and bias in the polygenic scores of complex diseases and traits in Finland. *Am. J. Hum. Genet.* **104**, 1169–1181 (2019). 31155286 10.1016/j.ajhg.2019.05.001 PMC6562021

[2] Berg, J. J. et al. Reduced signal for polygenic adaptation of height in UK Biobank. *Elife*10.7554/eLife.39725 (2019). 10.7554/eLife.39725 PMC6428572 30895923

[3] Sohail, M. et al. Polygenic adaptation on height is overestimated due to uncorrected stratification in genome-wide association studies. *Elife*10.7554/eLife.39702 (2019). 10.7554/eLife.39702 PMC6428571 30895926

[4] Morris, T. T., Davies, N. M., Hemani, G. & Smith, G. D. Population phenomena inflate genetic associations of complex social traits. *Sci. Adv.* **6**, eaay0328 (2020). 32426451 10.1126/sciadv.aay0328 PMC7159920

[5] Abdellaoui, A., Dolan, C. V., Verweij, K. J. H. & Nivard, M. G. Gene-environment correlations across geographic regions affect genome-wide association studies. *Nat. Genet.* **54**, 1345–1354 (2022). 35995948 10.1038/s41588-022-01158-0 PMC9470533

[6] Davies, N. M. et al. Within family Mendelian randomization studies. *Hum. Mol. Genet.* **28**, R170–R179 (2019). 31647093 10.1093/hmg/ddz204

[7] Howe, L. J. et al. Within-sibship genome-wide association analyses decrease bias in estimates of direct genetic effects. *Nat. Genet.* **54**, 581–592 (2022). 35534559 10.1038/s41588-022-01062-7 PMC9110300

[8] Trejo, S. & Domingue, B. W. Genetic nature or genetic nurture? Introducing social genetic parameters to quantify bias in polygenic score analyses. *Biodemography Soc. Biol.* **64**, 187–215 (2018). 31852332 10.1080/19485565.2019.1681257

[9] Warrington, N. M. et al. Maternal and fetal genetic effects on birth weight and their relevance to cardio-metabolic risk factors. *Nat. Genet.* **51**, 804–814 (2019). 31043758 10.1038/s41588-019-0403-1 PMC6522365

[10] Wu, Y. et al. Estimating genetic nurture with summary statistics of multigenerational genome-wide association studies. *Proc. Natl Acad. Sci. USA* **118**, e2023184118 (2021). 34131076 10.1073/pnas.2023184118 PMC8237646

[11] Kong, A. et al. The nature of nurture: effects of parental genotypes. *Science* **359**, 424–428 (2018). 29371463 10.1126/science.aan6877

[12] Weiner, D. J. et al. Polygenic transmission disequilibrium confirms that common and rare variation act additively to create risk for autism spectrum disorders. *Nat. Genet.* **49**, 978–985 (2017). 28504703 10.1038/ng.3863 PMC5552240

[13] SPARK Consortium SPARK: a US cohort of 50,000 families to accelerate autism research. *Neuron* **97**, 488–493 (2018). 29420931 10.1016/j.neuron.2018.01.015 PMC7444276

[14] Beaty, T. H. et al. A genome-wide association study of cleft lip with and without cleft palate identifies risk variants near MAFB and ABCA4. *Nat. Genet.* **42**, 525–529 (2010). 20436469 10.1038/ng.580 PMC2941216

[15] Cornelis, M. C. et al. The gene, environment association studies consortium (GENEVA): maximizing the knowledge obtained from GWAS by collaboration across studies of multiple conditions. *Genet. Epidemiol.* **34**, 364–372 (2010). 20091798 10.1002/gepi.20492 PMC2860056

[16] Bulik-Sullivan, B. et al. An atlas of genetic correlations across human diseases and traits. *Nat. Genet.* **47**, 1236–1241 (2015). 26414676 10.1038/ng.3406 PMC4797329

[17] Guan, J. et al. Family-based genome-wide association study designs for increased power and robustness. *Nat. Genet*. **57**, 1044–1052 (2025). 10.1038/s41588-025-02118-0 PMC11985344 40065166

[18] Grove, J. et al. Identification of common genetic risk variants for autism spectrum disorder. *Nat. Genet.* **51**, 431–444 (2019). 30804558 10.1038/s41588-019-0344-8 PMC6454898

[19] Sanchez-Roige, S. et al. CADM2 is implicated in impulsive personality and numerous other traits by genome- and phenome-wide association studies in humans and mice. *Transl. Psychiatry* **13**, 167 (2023). 37173343 10.1038/s41398-023-02453-y PMC10182097

[20] Casey, J. P. et al. A novel approach of homozygous haplotype sharing identifies candidate genes in autism spectrum disorder. *Hum. Genet.* **131**, 565–579 (2012). 21996756 10.1007/s00439-011-1094-6 PMC3303079

[21] Pasman, J. A. et al. The CADM2 gene and behavior: a phenome-wide scan in UK-Biobank. *Behav. Genet.* **52**, 306–314 (2022). 35867259 10.1007/s10519-022-10109-8 PMC9463269

[22] Lee, J. J. et al. Gene discovery and polygenic prediction from a genome-wide association study of educational attainment in 1.1 million individuals. *Nat. Genet.* **50**, 1112–1121 (2018). 30038396 10.1038/s41588-018-0147-3 PMC6393768

[23] Locke, A. E. et al. Genetic studies of body mass index yield new insights for obesity biology. *Nature* **518**, 197–206 (2015). 25673413 10.1038/nature14177 PMC4382211

[24] Morris, J. et al. Genetic variation in CADM2 as a link between psychological traits and obesity. *Sci. Rep.* **9**, 7339 (2019). 31089183 10.1038/s41598-019-43861-9 PMC6517397

[25] Sakic, B. et al. Unravelling the joint genetic architecture between psychiatic and insulin-related traits in the general population. Preprint at *medRxiv* https://doi.org/2024.10.04.24314905 (2024). 10.1016/j.nsa.2026.107017 PMC13312545 42375481

[26] Gusev, A. et al. Integrative approaches for large-scale transcriptome-wide association studies. *Nat. Genet.* **48**, 245–252 (2016). 26854917 10.1038/ng.3506 PMC4767558

[27] Lambert, S. A. et al. The Polygenic Score Catalog as an open database for reproducibility and systematic evaluation. *Nat. Genet.* **53**, 420–425 (2021). 33692568 10.1038/s41588-021-00783-5 PMC11165303

[28] Yu, Y. et al. Polygenic risk impacts PDGFRA mutation penetrance in non-syndromic cleft lip and palate. *Hum. Mol. Genet.* **31**, 2348–2357 (2022). 35147171 10.1093/hmg/ddac037 PMC9307317

[29] Xu, Y. et al. An atlas of genetic scores to predict multi-omic traits. *Nature* **616**, 123–131 (2023). 36991119 10.1038/s41586-023-05844-9 PMC10323211

[30] Yang, Y., Suzuki, A., Iwata, J. & Jun, G. Secondary genome-wide association study using novel analytical strategies disentangle genetic components of cleft lip and/or cleft palate in 1q32.2. *Genes* **11**, 1280 (2020). 33137956 10.3390/genes11111280 PMC7693579

[31] Schwender, H., Taub, M. A., Beaty, T. H., Marazita, M. L. & Ruczinski, I. Rapid testing of SNPs and gene–environment interactions in case–parent trio data based on exact analytic parameter estimation. *Biometrics* **68**, 766–773 (2012). 22150644 10.1111/j.1541-0420.2011.01713.x PMC3387527

[32] Spielman, R. S., McGinnis, R. E. & Ewens, W. J. Transmission test for linkage disequilibrium: the insulin gene region and insulin-dependent diabetes mellitus (IDDM). *Am. J. Hum. Genet.* **52**, 506–516 (1993). 8447318 PMC1682161

[33] Risch, R. & Merikangas, M. The future of genetic studies of complex human diseases. *Science* **273**, 1516–1517 (1996). 8801636 10.1126/science.273.5281.1516

[34] Ayres, K. L. & Curnow, R. N. Detecting non-multiplicative genotype relative risks from transmissions of parental alleles to affected children. *J. Hum. Genet.* **50**, 46–48 (2005). 15599640 10.1007/s10038-004-0217-5

[35] Clayton, D. & Jones, H. Transmission/disequilibrium tests for extended marker haplotypes. *Am. J. Hum. Genet.* **65**, 1161–1169 (1999). 10486335 10.1086/302566 PMC1288249

[36] Sham, P. C. & Curtis, D. An extended transmission/disequilibrium test (TDT) for multi-allele marker loci. *Ann. Hum. Genet.* **59**, 323–336 (1995). 7486838 10.1111/j.1469-1809.1995.tb00751.x

[37] Abecasis, G. R., Cookson, W. O. C. & Cardon, L. R. Pedigree tests of transmission disequilibrium. *Eur. J. Hum. Genet.* **8**, 545–551 (2000). 10909856 10.1038/sj.ejhg.5200494

[38] Schaid, D. J. Case-parents design for gene–environment interaction. *Genet. Epidemiol.* **16**, 261–273 (1999). 10096689 10.1002/(SICI)1098-2272(1999)16:3<261::AID-GEPI3>3.0.CO;2-M

[39] Umbach, D. M. & Weinberg, C. R. The use of case–parent triads to study joint effects of genotype and exposure. *Am. J. Hum. Genet.* **66**, 251–261 (2000). 10631155 10.1086/302707 PMC1288330

[40] Cordell, H. J., Barratt, B. J. & Clayton, D. G. Case/pseudocontrol analysis in genetic association studies: a unified framework for detection of genotype and haplotype associations, gene–gene and gene–environment interactions, and parent-of-origin effects. *Genet. Epidemiol.* **26**, 167–185 (2004). 15022205 10.1002/gepi.10307

[41] Mitchell, L. E. Differentiating between fetal and maternal genotypic effects, using the transmission test for linkage disequilibrium. *Am. J. Hum. Genet.* **60**, 1006–1007 (1997). 9106551 PMC1712475

[42] Weinberg, C. R., Wilcox, A. J. & Lie, R. T. A log-linear approach to case-parent-triad data: assessing effects of disease genes that act either directly or through maternal effects and that may be subject to parental imprinting. *Am. J. Hum. Genet.* **62**, 969–978 (1998). 9529360 10.1086/301802 PMC1377041

[43] Horvath, S., Xu, X. & Laird, N. M. The family based association test method: strategies for studying general genotype–phenotype associations. *Eur. J. Hum. Genet.* **9**, 301–306 (2001). 11313775 10.1038/sj.ejhg.5200625

[44] Lange, C. & Laird, N. M. On a general class of conditional tests for family-based association studies in genetics: the asymptotic distribution, the conditional power, and optimality considerations. *Genet. Epidemiol.* **23**, 165–180 (2002). 12214309 10.1002/gepi.209

[45] Lange, C., DeMeo, D., Silverman, E. K., Weiss, S. T. & Laird, N. M. PBAT: tools for family-based association studies. *Am. J. Hum. Genet.* **74**, 367–369 (2004). 14740322 10.1086/381563 PMC1181934

[46] Ding, Y. et al. Polygenic scoring accuracy varies across the genetic ancestry continuum. *Nature* **618**, 774–781 (2023). 37198491 10.1038/s41586-023-06079-4 PMC10284707

[47] Zhang, W. et al. Detecting gene-environment interaction for maternal exposures using case–parent trios ascertained through a case with non-syndromic orofacial cleft. *Front. Cell. Dev. Biol.* **9**, 621018 (2021). 33937227 10.3389/fcell.2021.621018 PMC8085423

[48] Khera, A. V. et al. Genetic risk, adherence to a healthy lifestyle, and coronary disease. *N. Engl. J. Med.* **375**, 2349–2358 (2016). 27959714 10.1056/NEJMoa1605086 PMC5338864

[49] Maas, P. et al. Breast cancer risk from modifiable and nonmodifiable risk factors among white women in the United States. *JAMA Oncol.* **2**, 1295–1302 (2016). 27228256 10.1001/jamaoncol.2016.1025 PMC5719876

[50] Garcia-Closas, M. et al. Common genetic polymorphisms modify the effect of smoking on absolute risk of bladder cancer. *Cancer Res.* **73**, 2211–2220 (2013). 23536561 10.1158/0008-5472.CAN-12-2388 PMC3688270

[51] Gaunt, T. R. et al. Systematic identification of genetic influences on methylation across the human life course. *Genome Biol.* **17**, 61–z (2016). 27036880 10.1186/s13059-016-0926-z PMC4818469

[52] Burgess, S. & Thompson, S. G. Multivariable Mendelian randomization: the use of pleiotropic genetic variants to estimate causal effects. *Am. J. Epidemiol.* **181**, 251–260 (2015). 25632051 10.1093/aje/kwu283 PMC4325677

[53] Bycroft, C. et al. The UK Biobank resource with deep phenotyping and genomic data. *Nature* **562**, 203–209 (2018). 30305743 10.1038/s41586-018-0579-z PMC6786975

[54] Schmidt, S. & Schaid, D. J. Potential misinterpretation of the case-only study to assess gene–environment interaction. *Am. J. Epidemiol.* **150**, 878–885 (1999). 10522659 10.1093/oxfordjournals.aje.a010093

[55] Falconer, D. S. & Mackay, T. F. C. *Introduction to Quantitative Genetics* (Addison Wesley Longman, 1996).

[56] An, J., Won, S., Lutz, S. M., Hecker, J. & Lange, C. Effect of population stratification on SNP-by-environment interaction. *Genet. Epidemiol.* **43**, 1046–1055 (2019). 31429121 10.1002/gepi.22250 PMC6829023

[57] Abdellaoui, A. et al. Genetic correlates of social stratification in Great Britain. *Nat. Hum. Behav.* **3**, 1332–1342 (2019). 31636407 10.1038/s41562-019-0757-5

[58] Veller, C. & Coop, G. M. Interpreting population- and family-based genome-wide association studies in the presence of confounding. *PLoS Biol.* **22**, e3002511 (2024). 38603516 10.1371/journal.pbio.3002511 PMC11008796

[59] Weissbrod, O. et al. Leveraging fine-mapping and multipopulation training data to improve cross-population polygenic risk scores. *Nat. Genet.* **54**, 450–458 (2022). 35393596 10.1038/s41588-022-01036-9 PMC9009299

[60] Schmilovich, Z. et al. Copy-number variants and polygenic risk for intelligence confer risk for autism spectrum disorder irrespective of their effects on cognitive ability. *Front. Psychiatry* **15**, 1369767 (2024). 38751416 10.3389/fpsyt.2024.1369767 PMC11094536

[61] Dai, X. et al. The role of circadian rhythms and sleep in the aetiology of autism spectrum disorder and attention-deficit/hyperactivity disorder: new evidence from bidirectional two-sample Mendelian randomization analysis. *Autism* **29**, 76–86 (2025). 38869021 10.1177/13623613241258546 PMC11656626

[62] Baranova, A. et al. Shared genetics between autism spectrum disorder and attention-deficit/hyperactivity disorder and their association with extraversion. *Psychiatry Res.* **314**, 114679 (2022). 35717853 10.1016/j.psychres.2022.114679

[63] Prive, F. et al. Portability of 245 polygenic scores when derived from the UK Biobank and applied to 9 ancestry groups from the same cohort. *Am. J. Hum. Genet.* **109**, 12–23 (2022). 34995502 10.1016/j.ajhg.2021.11.008 PMC8764121

[64] Zheutlin, A. B. et al. Penetrance and pleiotropy of polygenic risk scores for schizophrenia in 106,160 patients across four health care systems. *Am. J. Psychiatry* **176**, 846–855 (2019). 31416338 10.1176/appi.ajp.2019.18091085 PMC6961974

[65] Cai, N. et al. Minimal phenotyping yields genome-wide association signals of low specificity for major depression. *Nat. Genet.* **52**, 437–447 (2020). 32231276 10.1038/s41588-020-0594-5 PMC7906795

[66] Gui, Y. et al. Sex-specific genetic association between psychiatric disorders and cognition, behavior and brain imaging in children and adults. *Transl. Psychiatry*10.1038/s41398-022-02041-6 (2022). 10.1038/s41398-022-02041-6 PMC9418275 36028495

[67] Lahey, B. B. et al. Associations of polygenic risk for attention-deficit/hyperactivity disorder with general and specific dimensions of childhood psychological problems and facets of impulsivity. *J. Psychiatr. Res.* **152**, 187–193 (2022). 35752070 10.1016/j.jpsychires.2022.06.019 PMC10001434

[68] Chen, T. et al. Genomic insights for personalised care in lung cancer and smoking cessation: motivating at-risk individuals toward evidence-based health practices. *eBioMedicine* **110**, 105441 (2024). 10.1016/j.ebiom.2024.105441 PMC11583727 39520911

[69] Auton, A. et al. A global reference for human genetic variation. *Nature* **526**, 68–74 (2015). 26432245 10.1038/nature15393 PMC4750478

[70] Beaty, T. H. et al. Evidence for gene-environment interaction in a genome wide study of nonsyndromic cleft palate. *Genet. Epidemiol.* **35**, 469–478 (2011). 21618603 10.1002/gepi.20595 PMC3180858

[71] Wang, Z. PGS-TRI. *Zenodo*10.5281/zenodo.19189683 (2026).

[72] Wang, Z. PGS-TRI-Analysis. *Zenodo*10.5281/zenodo.19353771 (2026).

[73] Pebesma, E. & Bivand, R. *Spatial Data Science: With Applications in R* (Chapman and Hall/CRC, 2023).

[74] Pebesma, E. Simple features for R: standardized support for spatial vector data. *The R J.* **10**, 439–446 (2018).

