# Supplementary material for: Estimation of direct and indirect polygenic effects and gene–environment interactions using polygenic scores in case–parent trio studies
Source: Nat Genet. 2026 Jun 2;58(6):1237–47. doi: 10.1038/s41588-026-02601-2 (PMC13263142; doi:10.1038/s41588-026-02601-2)
Supplement: Supplementary file 2 — Reporting Summary [file 41588_2026_2601_MOESM2_ESM.pdf]

Corresponding author(s): Nilanjan Chatterjee

Last updated by author(s): Mar 28, 2026

Reporting Summary

Nature Portfolio wishes to improve the reproducibility of the work that we publish. This form provides structure for consistency and transparency in reporting. For further information on Nature Portfolio policies, see our Editorial Policies and the Editorial Policy Checklist.

Statistics

For all statistical analyses, confirm that the following items are present in the figure legend, table legend, main text, or Methods section.

n/a	Confirmed	
☐	☒	The exact sample size (*n*) for each experimental group/condition, given as a discrete number and unit of measurement
☐	☒	A statement on whether measurements were taken from distinct samples or whether the same sample was measured repeatedly
☐	☒	The statistical test(s) used AND whether they are one- or two-sided<br>*Only common tests should be described solely by name; describe more complex techniques in the Methods section.*
☐	☒	A description of all covariates tested
☐	☒	A description of any assumptions or corrections, such as tests of normality and adjustment for multiple comparisons
☐	☒	A full description of the statistical parameters including central tendency (e.g. means) or other basic estimates (e.g. regression coefficient) AND variation (e.g. standard deviation) or associated estimates of uncertainty (e.g. confidence intervals)
☐	☒	For null hypothesis testing, the test statistic (e.g. *F*, *t*, *r*) with confidence intervals, effect sizes, degrees of freedom and *P* value noted<br>*Give P values as exact values whenever suitable.*
☒	☐	For Bayesian analysis, information on the choice of priors and Markov chain Monte Carlo settings
☐	☒	For hierarchical and complex designs, identification of the appropriate level for tests and full reporting of outcomes
☐	☒	Estimates of effect sizes (e.g. Cohen's *d*, Pearson's *r*), indicating how they were calculated

Our web collection on statistics for biologists contains articles on many of the points above.

Software and code

Policy information about availability of computer code

Data collection	No software was used for data collection as study involves analysis of existing dataset.
Data analysis	All custom codes used to perform data analysis relevant to this paper, including software codes of PGS-TRI, the 4 sets of simulation studies including using the UK Biobank and SNIPAR, data cleaning and preparation using reference data set from the 1000 Genome + HGDP Project, data analyses using trio data for the SPARK consortium and GENEVA study, as well as the transcriptome-wide and metabolome-wide association analyses using OMICSPRED and GTEx v8, the sensitivity analyses using genotypic TDT test, the codes of figures in the manuscript are available at https://github.com/ziqiaow/PGS-TRI-Analysis, and at Zenodo with https://doi.org/10.5281/zenodo.19353772. The software tool of the proposed method, example codes, and documentations are available at https://github.com/ziqiaow/PGS.TRI and at Zenodo with https://doi.org/10.5281/zenodo.19339894. The majority of our statistical analysis was performed using R 4.2.1, and R packages include ggplot2 3.5.1, data.table 1.14.4, ggpubr 0.6.0, ggsci 2.9, tidyverse 1.3.2, dplyr 1.11, patchwork 1.1.2, gridExtra 2.3, viridis 0.6.2, RColorBrewer 1.1-3, ggrepel 0.9.2, latex2exp 0.9.6, readr 2.1.3, reshape2 1.4.4, sf 1.0-16, ggfortify 0.4.16, scales 1.3.0, cowplot 1.1.1, fastDummies 1.6.3, devtools 2.4.4, stringr 1.4.0. The publicly available summary-level statistics and analysis relevant to analyzing genotype data were performed by PLINK 2.0 and PLINK 1.9. The genotypic TDT test was performed using trio 3.34.0. The analyses of polygenic TDT test and case-only analyses was performed using author's own developed codes, available at https://github.com/ziqiaow/PGS.TRI. The imputation of genotype data in the SPARK consortium was based on the TOPMed Imputation server; and the preprocessing of genotypic data in the GENEVA study was available in previous publication (DOI: 10.3389/fcell.2021.621018).

For manuscripts utilizing custom algorithms or software that are central to the research but not yet described in published literature, software must be made available to editors and reviewers. We strongly encourage code deposition in a community repository (e.g. GitHub). See the Nature Portfolio guidelines for submitting code & software for further information.

Data

Policy information about availability of data

All manuscripts must include a data availability statement. This statement should provide the following information, where applicable:

- Accession codes, unique identifiers, or web links for publicly available datasets
- A description of any restrictions on data availability
- For clinical datasets or third party data, please ensure that the statement adheres to our policy

Summary statistics of the results of all PGS-TRI and pTDT analyses in the SPARK consortium and GENEVA study, irrespective of significance level are available in supplemental tables of the manuscript. For individual-level genetic and phenotypic data, GENEVA datasets are available in dbGaP at https://www.ncbi.nlm.nih.gov through dbGaP accession number phs000094.v1.p1. For individual-level phenotypic and genetic data on SFARI Base, approved researchers can obtain the SPARK population dataset described in this study by applying at https://base.sfari.org. GRCh37 and GRCh38 reference genome data from Phase-3 1000 Genome Project is available from https://www.internationalgenome.org/data. PC-based ancestry information and CRCh38 reference genome data (the gnomAD v3.1.2) from 1000 Genome + HGDP Project is available from https://gnomad.broadinstitute.org/downloads#v3. The detailed population information is extracted from IGSR: the International Genome Sample Resource: https://www.internationalgenome.org/data-portal/sample. Access to UK Biobank individual level data can be requested from https://www.ukbiobank.ac.uk/enable-your-research/apply-for-access. The GTEx v8 genetic scores for gene expression of brain tissues are downloaded from http://gusevlab.org/projects/fusion/#gtex-v8-multi-tissue-expression. OMICSPRED genetic scores are downloaded from https://www.omicspred.org/.

Research involving human participants, their data, or biological material

Policy information about studies with human participants or human data. See also policy information about sex, gender (identity/presentation), and sexual orientation and race, ethnicity and racism.

Reporting on sex and gender	The analyses was based on existing data of case-parent trios in the SPARK consortium and GENEVA study. The sex of children were considered in the GxE interaction analyses and results were reported in the main manuscript and in the supplemental tables.
Reporting on race, ethnicity, or other socially relevant groupings	For the SPARK consortium data, the proband ancestry estimates were generated by Regenron, with their own joint genotyping data and HapMap3 database as a reference panel, using the HapMap populations and ancestry superclasses below.<br><br>HapMap3 Populations:<br>● ASW African ancestry in southwest United States<br>● CEU Utah residents (CEPH) with northern and western European ancestry<br>● CHB Han Chinese in Beijing<br>● CHD Chinese in Denver<br>● GIH Gujarati Indians in Houston<br>● JPT Japanese in Tokyo<br>● LWK Luhya in Webuye, Kenya<br>● MEX Mexican ancestry in Los Angeles<br>● MKK Maasai in Kinyawa, Kenya<br>● TSI Toscana in Italy<br>● YRI Yoruba in Ibadan, Nigeria<br><br>Ancestry Superclasses:<br>● AFR African<br>● AMR Americas<br>● EAS East Asian<br>● EUR European<br>● SAS South Asian<br><br>We summarized the number of children, mothers and fathers in each ancestry superclass used in the analysis in the Supplemental Table S1 of our manuscript.<br><br>For GENEVA study, the case–parent trios were recruited largely through surgical treatment centers by multiple investigators from Europe (Norway), the United States (Iowa, Maryland, Pennsylvania, and Utah) and Asia (People's Republic of China, Taiwan, South Korea, Singapore, and the Philippines) (Beaty et al., 2010, 2011; Leslie et al., 2017). The race of each proband was determined by self-reported race.
Population characteristics	The detailed population characteristics of ancestry groups of probands in the case-parent trios, the number of orofacial clefts subtypes in the analysis, and the maternal environmental exposures related to GxE interactions analyses are available in Supplemental Tables S1 and S11-S12.
Recruitment	The current research only used data from available databases and biobanks, it did not involve the recruitment of human participants.
Ethics oversight	The research protocol for GENEVA was approved by the Institutional Review Boards (IRBs) at the Johns Hopkins Bloomberg School of Public Health and at each participating recruitment site. All authors with data access have the CITI certificates for human research and Information Privacy and Security (IPS) at Johns Hopkins Bloomberg School of Public Health.

Note that full information on the approval of the study protocol must also be provided in the manuscript.

Field-specific reporting

Please select the one below that is the best fit for your research. If you are not sure, read the appropriate sections before making your selection.

☒ Life sciences ☐ Behavioural & social sciences ☐ Ecological, evolutionary & environmental sciences

For a reference copy of the document with all sections, see nature.com/documents/nr-reporting-summary-flat.pdf

Life sciences study design

All studies must disclose on these points even when the disclosure is negative.

Sample size	We analyzed existing data from the SPARK consortium, after quality control, includes families with ASD-proband of 1,235 African, 2,410 Americas, 442 East Asian, 13,668 European, and 628 South Asian case-parent trios (in total 18,383 trios/55,149 participants). More details of the sample size is available in Supplemental Table S1. While no a priori sample size calculations were performed as this is an analysis of existing data, we note that our study is the first of its kind for the non-European case-parent trio analyses. For the GENEVA study, we analyzed existing data including 1,126 East Asian and 778 European case-parent trios (in total 1,904 trios/5,712 participants).
Data exclusions	We excluded participants without genotype data and missing phenotype data from the analyses. We further excluded siblings of case-parent trios to make sure the analyses only included complete unrelated case-parent trio families.
Replication	All analysis scripts and code are publicly available at https://github.com/ziqiaow/PGS-TRI-Analysis to facilitate reproducible computational research.
Randomization	Not applicable. This is an observational study.
Blinding	Not applicable. This is an observational study.

Reporting for specific materials, systems and methods

We require information from authors about some types of materials, experimental systems and methods used in many studies. Here, indicate whether each material, system or method listed is relevant to your study. If you are not sure if a list item applies to your research, read the appropriate section before selecting a response.

Materials & experimental systems

n/a	Involved in the study	
☒	☐	Antibodies
☒	☐	Eukaryotic cell lines
☒	☐	Palaeontology and archaeology
☒	☐	Animals and other organisms
☒	☐	Clinical data
☒	☐	Dual use research of concern
☒	☐	Plants

Methods

n/a	Involved in the study	
☒	☐	ChIP-seq
☒	☐	Flow cytometry
☒	☐	MRI-based neuroimaging

Plants

Seed stocks	*Report on the source of all seed stocks or other plant material used. If applicable, state the seed stock centre and catalogue number. If plant specimens were collected from the field, describe the collection location, date and sampling procedures.*
Novel plant genotypes	*Describe the methods by which all novel plant genotypes were produced. This includes those generated by transgenic approaches, gene editing, chemical/radiation-based mutagenesis and hybridization. For transgenic lines, describe the transformation method, the number of independent lines analyzed and the generation upon which experiments were performed. For gene-edited lines, describe the editor used, the endogenous sequence targeted for editing, the targeting guide RNA sequence (if applicable) and how the editor was applied.*
Authentication	*Describe any authentication procedures for each seed stock used or novel genotype generated. Describe any experiments used to assess the effect of a mutation and, where applicable, how potential secondary effects (e.g. second site T-DNA insertions, mosiacism, off-target gene editing) were examined.*
