# Supplementary material for: Estimation of direct and indirect polygenic effects and gene–environment interactions using polygenic scores in case–parent trio studies
Source: Nat Genet. 2026 Jun 2;58(6):1237–47. doi: 10.1038/s41588-026-02601-2 (PMC13263142; doi:10.1038/s41588-026-02601-2)
Supplement: Supplementary file 3 — Peer Review File [file 41588_2026_2601_MOESM3_ESM.pdf]

nature portfolio

Peer Review File

Estimation of Direct and Indirect Polygenic Effects and Gene-Environment Interactions using Polygenic Scores in Case-Parent Trio Studies

Corresponding Author: Dr Nilanjan Chatterjee

A version of this paper was originally rejected for publication by Nature Genetics, however that decision was reconsidered after appeal by the authors.

Version 0:

Decision Letter:

9th Jan 2025

Dear Dr Chatterjee,

Your Article entitled "Estimation of Direct and Indirect Polygenic Effects and Gene-Environment Interactions using Polygenic Scores in Case-Parent Trio Studies" has now been seen by 3 referees, whose comments are attached. In the light of their advice we have decided that we cannot offer to publish your manuscript in Nature Genetics, at least in its present form.

While the referees find your work of some interest, they raise serious concerns about the strength of the novel conclusions that can be drawn at this stage. We feel that these reservations are sufficiently important as to preclude publication of this study in Nature Genetics.

In the future, if further experimental data allow you to fully address these criticisms, you may contact us to discuss a potential appeal of our decision (unless, of course, something similar has by then been accepted at Nature Genetics or appeared elsewhere). This includes submission or publication of a portion of this work someplace else. The required new experiments and data include, but are not limited to those detailed here. We hope you understand that until we have read the revised manuscript in its entirety we cannot promise that it will be sent back for peer review.

Otherwise, we hope that you find our referees' comments helpful when preparing your manuscript for resubmission elsewhere.

Although we cannot publish your paper, it may be appropriate for another journal in the Nature Portfolio. If you wish to explore the journals and transfer your manuscript please use our manuscript transfer portal. You will not have to re-supply manuscript metadata and files, unless you wish to make modifications. For more information, please see our manuscript transfer FAQ page.

Sincerely,

Wei Li, PhD
Senior Editor
Nature Genetics
www.nature.com/ng

Reviewers' Comments:

Reviewer #1 (Remarks to the Author):

The authors extend prior methods for evaluating the association of polygenic risk scores (PRS) with binary disease status when sampling diseased cases and their parents. The strengths of this paper are the innovative statistical methods that address scientific questions about direct and indirect effects of PRS on a child's risk of disease. The benefits of the case-parent design, in terms of controlling for population stratification, are well known. The insights this paper provides, and the appropriate methods to do so, are the need to appropriately scale estimates of genetic effects to avoid bias, the ability to distinguish between direct and indirect effects of parental PRS on child's disease risk; the ability to evaluate whether the maternal and paternal indirect effects are the same; and the ability to estimate the interaction of direct genetic effects with environmental risk factors. The simulations demonstrate that the statistical methods control the false-positive rate and are unbiased, and perform as expected. The applications to the autism spectrum data (ASD) and the orofacial cleft data (OFC) nicely demonstrate how the methods can be used, and in particular provide some novel findings (e.g., maternal mediated indirect effects on risk of ASD in children; SNP x smoking interaction on OFC). The discussion is well-balanced in terms of highlighting the novel contributions of both statistical methods and results from the ASD and OFC analyses, as well as limitations of the metehods. Overall, this paper is well written with appropriate tables and figures. I have a few relatively minor questions.

Because logistic regression is commonly used for binary disease status, I think it would help the reader if you could explain why you use log-linear regression instead of logistic regression.

I think the justification of the assumption of fine-scale homogenous families needs more clarity. The authors state that their methods can account for assortative mating, but the assumption of random mating within the fine-scale homogenous populations seems unrealistic, as an attempt to circumvent the association between parents due to assortative mating. More clarity on this justification and why it accounts for assortative mating in real populations is needed. This is also relevant to the gene-environment independence assumption.

On line 18 of page 8, can you give an intuitive explanation of why the estimate of variance has '2" in the denominator? The rest of the equation makes sense, based on sample variance estimate.

On page 11, lines 21-21, state that PGS are standardized by ancestry-specific standard errors based on 1000 genome reference. How does this approach to standardization, which relies on self-reported ancestry, compare with the continuous PC projection approach available in the pgsc-calc (PGS Catalog)? Does your approach account for when parents are of mixed ancestry?

Please explain the purpose of "Extended Figures". Are these to be in the supplement? If in the main article, there seem to be too many figures.

Reviewer #1 (Remarks on code availability):

I briefly reviewed the code, vignette, and help files. I installed the R package and ran the simulation in the vignette. All seemed to work fine.

Reviewer #2 (Remarks to the Author):

What are the major claims of the paper?

That family based samples and PGS can be used to estimate direct and indirect effects.

Are they novel and do you think that they represent a significant advance in the field?

There has been a lot of interest in family-based samples for estimating indirect effects. For example, Kong et al. (2018), used directly and indirectly transmitted polygenic scores to investigate indirect effects in a range of traits in deCODE. That was a bigger sample, and covered more traits. For Autism, Weiner et al. (2017) used polygenic transmission disequilibrium tests to investigate the transmission of various traits in 6,454 families. These are both referenced in the paper.

The key contribution claimed for this study is that the previously cited papers did not present their results on an easily interpretable risk scale. This is a fairly standard rescaling of coefficients and doesn't represent a particularly major advance. E.g. see Tan et al. (2024) or Nivard et al (2024).

Tan et al. 2024. 'Family-GWAS Reveals Effects of Environment and Mating on Genetic Associations'.
Nivard et al 2024. 'More than Nature and Nurture, Indirect Genetic Effects on Children's Academic Achievement Are

Consequences of Dynastic Social Processes'. Nature Human Behaviour 8(4):771–78. doi: 10.1038/s41562-023-01796-2.

Is the work convincing, and if not, what further evidence would be required to strengthen the conclusions?

The results are not convincing, because the authors' central claim, that the indirect effects can be estimated from the polygenic scores is not demonstrated by their simulations or derivations.

The key challenge is differentiating between effects due to assortative mating and population structure versus indirect effects. The authors essentially assume this problem away in their simulations without justifying why. They also do not address selection and collider bias. As a result, their simulations and empirical examples are not convincing.

On a more subjective note, do you feel that the paper will influence thinking in the field and be of interest to a wider community of researchers?

This paper is unlikely to have a major influence on the field. The data and simulations do not tackle the key challenges above, or demonstrate that the central claim made by the paper (indirect effects can be estimated using trios) is likely to hold.

o Are you satisfied that all data and source code needed to reproduce the results of the paper have been made available?

The code is publicly available.

o Are you satisfied that the results can be replicated using the code/software and dataset provided in the study?

Yes.

o Was the code sufficiently documented to allow another researcher to follow the algorithm?

Yes.

O Can the software be run on a widely available operating system?

Yes

o To your knowledge, do available tools or software exist that perform in a similar way to the reported software?

No.

Major comments

1. It would be worth updating the simulation to simulate the transmission of individual genotypes rather than the continuous scores. This simplification of the simulation means it omits the key point of randomized transmission of variants from parents to offspring.

2. The current paper doesn't address selection or collider bias - this is another explanation for the results and can cause bias, even in samples of related individuals.

3. It would be useful to prove how it is possible to differentiate indirect effects due to the effects of parental phenotype and the effects of an environmental confounder or assortative mating. The following statement:

"We conceptualize a sampling mechanism where for each family sampled, we assume it belongs to a unique subpopulation of "homogeneous" families with distinct genetic ancestry and trait characteristics influencing assortative mating. The number of these subpopulations can be arbitrarily large, making each subpopulation at an extremely fine level. It is assumed random mating occurs within these homogeneous fine-level subpopulations, implying the PGS correlation between partners is 0 within these subpopulations, but not necessarily across them."

This approach is not explained in sufficient detail in the rest of the paper. The UK Biobank example does not adequately assess the robustness of this method to bias from assortative mating or population stratification. This should be assessed via simulations that induce assortment/confounding via structure into DGP.

4. Power - it would be worth running simulations to assess the relative power of these approaches as a function of R^2 of the trait of interest, and sample size.

5. ASD GWAS samples are small. How well did it predict ASD in the sample?

6. Have the authors ruled out gene-environment correlation when conducting the GxE interaction tests?

Minor

Could you confirm whether there was any sample overlap between the GWAS used to generate the scores and the samples

used in your analysis?
page 3 line 22 “randomly sampled families and are not” - could you confirm that these studies were randomly sampled families?
Page 4 line 24 “We obtain transmission-based estimates of effect sizes for PGS underlying these two traits and show that they are comparable to corresponding effect sizes reported in prior studies, primarily conducted based on unrelated cases and controls”. Could present a direct comparison of your estimates with those from prior studies?
Page 4 line 29 “For both ASD and OFCs, we examine PGSxE interactions for several known maternal risk factors”. How did the results compare to estimates from other studies? To what extent was the variance reduced.
Page 6 line 16 “Finally, we assume $EiC \perp (PGSiC, PGSiM, PGSiF)$, but allow the distribution $\boldsymbol{EiC}$ to remain unspecified. From a population perspective, this assumption can again be viewed as “gene-environment” independence within highly homogenous subpopulations” and “For the investigation of gene-environment interactions, we incorporate a binary ($E1$) and a continuous ($E2$) variable in the disease model. We allowed complex inter-relationships among baseline disease risk (αi), PGS mean (μi) and exposure means ($\gamma i1$ and $\gamma i2$) across families,”
-How does the model account for the variance in E between families, which impacts GxE. Why do you assume gene-environment independence, is this assumption plausible? To what extent are you simply assuming confounding away in your models and simulations?
Page 8 - line 22 - Why do you make this modification?
Page 9 line 19 - “Known to be highly confounded with environmental factors” - did these studies prove which of pop strat, indirect effects and assortment confounds and explains these associations?
Page 9 line 21 - Why did you create your score based on PGS002012? Really the key parameter here is R^2, and the LD structure etc, is not terribly relevant.
Page 9 - how did you simulate assortative mating? E.g. what functions and code?
Page 9 - Mendelian transmission - could you simulate the transmission of genotype rather than the transmission of the normally distributed continuous liability?
Page 10 line 19. “As a negative control, we used body mass index (BMI)”
Why is BMI considered a negative control? Many of these conditions are associated with BMI, especially depression, SCZ (positively) and ED (negatively). Why not height?
Page 10 - line 22 - “We 22 standardized all of the PGSs by ancestry-specific standard errors obtained from the 1000 23 Genomes (1000G) Phase 3 Project.24” How did you standardize the PGS? By what SE? How does this allow for interpreting the underlying risk parameters on the standard risk scale?
Page 10 - line 29 - Random transmission of the ASD score should ensure independence of the ASD score and the OMICSPRED gene expression and metabolite PRSs. If you do find a direct association here, then this suggests that your data may suffer from selection and collider bias.
Page 13 - line 21 - “low power and perhaps lack of 21 transportability of the PGS to non-EUR populations (Table S5).” Did you find any evidence that the effect of the PGS on the trait in question (if measured) in these populations differed?
Page 13 line 29 - How can you differentiate the maternal effects from assortment on that trait in the parental generation? Somewhere it’d be good to include a directed acyclic graph encoding the assumptions you’re making, and potentially comparing them to other approaches such as sibling regression.
Page 17 - line 10 - the intuition for why this rescaling is necessary is not clearly explained in the paper. I agree rescaling is necessary, but why by the within family variance in the PGS?
Page 18 - line 5 - but surely the maternal BMI score is far better powered than the metabolite scores? Larger GWAS, but perhaps it explains less of the variance in BMI than the metabolites? This would be a very nice point to make (more proximal measures like metabolites can provide more accurate results with smaller samples).
Page 19 - line 11 - “not well predicted” - MR analyses would test this association, either using summary data or IPD, can you provide some similar guidance here, is it possible to test whether the PGS sufficiently predict your exposure?

Reviewer #3 (Remarks to the Author):

Co-Review with Reviewer #2

Version 1:

Decision Letter:

IMPORTANT: Please note the reference number: NG-A66988R-Z Chatterjee. This number must be quoted whenever you communicate with us regarding this paper.

14th Jul 2025

Dear Dr. Chatterjee,

Thank you for your message of asking us to reconsider our decision on your manuscript "Estimation of Direct and Indirect Polygenic Effects and Gene-Environment Interactions using Polygenic Scores in Case-Parent Trio Studies". I have now discussed the points of your letter with my colleagues, including with the Chief Editor. And we think that you have some valid points.

However, at this stage we will need you to provide a point-by-point “Response to referees” document detailing the changes you have made and your response to the points raised. The editorial team may decide to send the revised manuscript back to the original reviewers.

At this stage we will need you to upload:

1) a point-by-point “Response to referees” document, detailing how you addressed each referee comment. If no action was taken to address a point, you must provide a compelling argument. This response will be sent back to the referees along with the revised manuscript.

2) a copy of the manuscript in MS Word .docx format.

3) a revised version of the Reporting Summary: https://www.nature.com/documents/nr-reporting-summary.pdf
(Here you can read about the role of the Reporting Summary in reproducible science:
https://www.nature.com/news/announcement-towards-greater-reproducibility-for-life-sciences-research-in-nature-1.22062)

When preparing a revision, please ensure that it fully complies with our editorial requirements for format and style; details can be found in the Guide to Authors on our website (http://www.nature.com/ng/).

Please use the link below to be taken directly to the site and view and revise your manuscript:

Link Redacted

With kind wishes,
Wei

Wei Li, PhD
Senior Editor
Nature Genetics
www.nature.com/ng

Version 2:

Decision Letter:

4th Nov 2025

Dear Dr Chatterjee,

Your Article, "Estimation of Direct and Indirect Polygenic Effects and Gene-Environment Interactions using Polygenic Scores in Case-Parent Trio Studies" has now been seen by 4 referees. You will see from their comments below that while they find your work of interest, some important points are raised. We are interested in the possibility of publishing your study in Nature Genetics, but would like to consider your response to these concerns in the form of a substantially revised manuscript before we make a final decision on publication.

To guide the scope of the revisions, the editors discuss the referee reports in detail within the team, including with the chief editor, with a view to identifying key priorities that should be addressed in revision. In this case, Reviewer #4 raises major concerns that need to be addressed or clarified. Wei ask that you address their comments sd thoroughly as possible with appropriate revisions. Please do not hesitate to get in touch if you would like to discuss these issues further.

We therefore invite you to revise your manuscript taking into account all reviewer and editor comments. Please highlight all changes in the manuscript text file. At this stage we will need you to upload a copy of the manuscript in MS Word .docx or similar editable format.

We are committed to providing a fair and constructive peer-review process. Do not hesitate to contact us if there are specific requests from the reviewers that you believe are technically impossible or unlikely to yield a meaningful outcome.

When revising your manuscript:

*1) Include a “Response to referees” document detailing, point-by-point, how you addressed each referee comment. If no action was taken to address a point, you must provide a compelling argument. This response will be sent back to the referees along with the revised manuscript.

*2) If you have not done so already please begin to revise your manuscript so that it conforms to our Article format instructions, available
here.
Refer also to any guidelines provided in this letter.

*3) Include a revised version of any required Reporting Summary: https://www.nature.com/documents/nr-reporting-summary.pdf
It will be available to referees (and, potentially, statisticians) to aid in their evaluation if the manuscript goes back for peer review.
A revised checklist is essential for re-review of the paper.

Please be aware of our guidelines on digital image standards.

EXTENDED DATA FIGURES

When re-submitting your manuscript, please ensure that any supplementary figures and tables that are crucial to the manuscript’s conclusions are converted into Extended Data figures and tables to increase visibility of these data. Extended Data figures and tables are online-only (present in the online PDF and full-text HTML versions of the paper), peer-reviewed display items that provide essential background to the article but are not included in the main article due to space constraints. A maximum of ten Extended Data display items (figures and tables) is permitted.

Please use the link below to submit your revised manuscript and related files:

Link Redacted

Note: This URL links to your confidential home page and associated information about manuscripts you may have submitted, or that you are reviewing for us. If you wish to forward this email to co-authors, please delete the link to your homepage.

We hope to receive your revised manuscript within 8 to 12 weeks. If you cannot send it within this time, please let us know.

Please do not hesitate to contact me if you have any questions or would like to discuss these revisions further.

Nature Genetics is committed to improving transparency in authorship. As part of our efforts in this direction, we are now requesting that all authors identified as ‘corresponding author’ on published papers create and link their Open Researcher and Contributor Identifier (ORCID) with their account on the Manuscript Tracking System (MTS), prior to acceptance. ORCID helps the scientific community achieve unambiguous attribution of all scholarly contributions. You can create and link your ORCID from the home page of the MTS by clicking on ‘Modify my Springer Nature account’. For more information please visit please visit www.springernature.com/orcid.

We look forward to seeing the revised manuscript and thank you for the opportunity to review your work.

Sincerely,

Wei Li, PhD
Senior Editor
Nature Genetics
www.nature.com/ng

Reviewers' Comments:

Reviewer #1 (Remarks to the Author):

The authors' replies and revisions addressed my questions and concerns. Their careful and thorough evaluations,

combining statistical/population genetic theory with simulations and data analyses, provide convincing evidence that their analytic methods are robust and can provide novel insights about the role of genetics for complex traits, yet with acknowledged limitations. Expanding the sample size for ASD increased power and address a timely issue. Overall, the presented work is clear and impactful.

Reviewer #1 (Remarks on code availability):

Code was reviewed at first submission of article.

Reviewer #2 (Remarks to the Author):

A very comprehensive response, thank you for considering our comments.

Reviewer #3 (Remarks to the Author):

Thank you to the authors for their comprehensive responses to the feedback and comments. They have appropriately addressed all feedback and the revised manuscript, with additional analyses and large increase in sample size, has strengthened the paper.

Reviewer #4 (Remarks to the Author):

The manuscript by Dr. Wang and colleagues present PGS-TRI, a method that leverages genetic and phenotypic data from trios (both parents and an child) to estimate direct, indirect, and gene-environment effects. The major benefit of trios is robustness to population stratification and assortative mating, when analyzing the child's phenotype conditional on the parental genotypes. This has been appreciated for many years and many statistical methods have been developed around this idea, which is a blueprint this manuscript follows.

Unfortunately, I found the manuscript hard to follow and the analyses scattered across many datasets, datatypes, and hypotheses. I do not think this manuscript is a good candidate for publication in Nature Genetics. In my view, the major weakeness are: 1) limited applicability of the method and 2) limited interesting findings when the method is applied to the largest datasets the method is applicable to. I highlight major issues below:

Limited applicability of the method:
While trio data is very powerful, the sample size has remained small. It may be true that more data is coming, but as it stands I do not think the method is of interest to the broad readership of Nature Genetics.

Limited interesting findings:
While gene-environment interactions are a primary interest of the method (it is in the title of the paper), the authors do not find convincing evidence (i.e. strong p-values) in real data for GxE for the two main disease traits they study, autism spectrum disorder and orofacial cleft. The authors claim that a major novelty of their method is the GxE component, but in their application of the method they do not find significant hits (see below). The authors do find evidence of direct genetic effects, but these do not seem novel. The authors find evidence for indirect genetic effects, although I am not convinced by them (see below). Other findings have been previously reported, for example the TRAF3IP3 association with OFCs (PMID: 33137956, Ref. 46 in manuscript). (Similar for CADM2). Taken together, the findings are of limited interest.

Lack of rigorous statistical control:
The authors report nominal significance in their main figures and the main description of the results largely excludes reporting p-values. This is not acceptable. Correction for multiple hypothesis testing is standard in the field. This has implications for the findings reported in the mauscript: In Table S7, for indirect genetic effects I see 5 tests that pass a Bonferonni threshold in the Cross-Population test, while the authors report 10 significant tests.

PGS x Context analysis:
I do not think this analysis tells us anything about the genetic architecture of autism. I could not find an equation corresponding to what the p-value < 1e-4 in Figure 4C. I do not understand the motivation for this analysis in this manuscript, as ancestry is not directly relevant for PGS-TRI. Further, the interaction itself is not interesting because many scenarios could lead to significant PGS x Context interactions. At a minimum, the authors should convice the reader that this is not due to scale effects. For example, consider the following R code that models additive effects on the liability scale, but leads to a significant PGS x Context term in a regression:

```
> N=1000
> PGS = rnorm(N)
> Context = rnorm(N)
> L = PGS + Context
> Disease = L < 3
> summary(lm(Disease ~ PGS*Context))
```

Can the authors rule out a model like this?

Choice of traits analyzed:
I could not find a justification for the set of traits analyzed in this manuscript, which seem random. In addition, the TWAS analysis is not well-motivated.

Selection bias results and differences in male female ascertainment (Supplementary section 2.4).
The authors show strong bias in the method for detecting indirect genetic effects when there is differential selection bias across the parents, which is sensible given their test statistic is testing for differences in PGS between the parents. However, the authors do not do any analyses testing the sensitivity of their Figure 4B results to this kind of bias or explain why we shouldn't worry about that kind of bias.

Less Major Issues:
- Line 156: "We assume within a family, PGS for the parents are independently distributed" Why is this assumption necessary? Does assortative mating lead to issues because of this assumption?
- Line 205: How can you get accurate estimates of the within-family variance given that trios only contain 3 data points?
- Citation in the response to reviewers of a personal communication to Dr. Alexander Young is not convincing.
- Line 450: "indicating polygenic risk and environmental factors generally act multiplicatively on the risk of ASD." Have the authors ruled out lack of statistical power as an explanation?
- Extended Figure 5B: inflated false positive rate in null simulations. On line 418, the authors claim that PGS-TRI remains unbiased in the presence of assortative mating, but this appears at odds with Extended Figure 5B, which shows bias in the presence of assortative mating.

Version 3:

Decision Letter:

Our ref: NG-A66988R2

6th Feb 2026

Dear Dr. Chatterjee,

Thank you for submitting your revised manuscript "Estimation of Direct and Indirect Polygenic Effects and Gene-Environment Interactions using Polygenic Scores in Case-Parent Trio Studies" (NG-A66988R2). It has now been seen by the original referees and their comments are below. The reviewers find that the paper has improved in revision, and therefore we'll be happy in principle to publish it in Nature Genetics, pending minor revisions to comply with our editorial and formatting guidelines.

We are now performing detailed checks on your paper and will send you a checklist detailing our editorial and formatting requirements soon. Please do not upload the final materials and make any revisions until you receive this additional information from us.

Thank you again for your interest in Nature Genetics Please do not hesitate to contact me if you have any questions.

Sincerely,

Wei Li, PhD
Senior Editor
Nature Genetics
www.nature.com/ng

Reviewer #4 (Remarks to the Author):

The authors have adequately addressed my concerns.

Open Access This Peer Review File is licensed under a Creative Commons Attribution 4.0 International License, which permits use, sharing, adaptation, distribution and reproduction in any medium or format, as long as you give appropriate credit to the original author(s) and the source, provide a link to the Creative Commons license, and indicate if changes were made.
In cases where reviewers are anonymous, credit should be given to 'Anonymous Referee' and the source.
The images or other third party material in this Peer Review File are included in the article's Creative Commons license, unless indicated otherwise in a credit line to the material. If material is not included in the article's Creative Commons license and your intended use is not permitted by statutory regulation or exceeds the permitted use, you will need to obtain permission directly from the copyright holder.
To view a copy of this license, visit https://creativecommons.org/licenses/by/4.0/

Response to referees

We are grateful to the reviewers for their insightful comments. We have revised the manuscript accordingly and provided a detailed response to each comment below. In summary, we have undertaken substantial new work—including expanded data analyses, simulations, and methodological derivations—to strengthen our study. We believe these efforts robustly demonstrate the utility and novelty of our method for analyzing polygenic risk scores (PGS) in case-parent trio studies. Key updates include:

1. **Major expansion of the analysis of Autism Spectrum Disorder Trios**
   We have increased the sample size for our ASD study from 1,517 to 18,383 trios.

   - This allowed a robust characterization of direct effects of established ASD polygenic scores across ancestry groups and along the genetic ancestry continuum using transmission-based statistics. We show that PGS performance declines continuously with increasing genetic distance between the training and target samples—a pattern previously observed in population-based studies (Ding et al., Nature, 2023). To our knowledge, this is the first family-based analysis to demonstrate this effect, indicating it is not merely an artifact of population stratification.

   - We now detect highly significant indirect effects of parental PGS for several additional traits on ASD risk—supporting existing hypotheses (e.g., causal effect of maternal obesity) and revealing novel associations (e.g., maternal ADHD PGS).

2. **New simulations**
   We conducted extensive simulations to assess robustness to assortative mating and selection bias.

   - Using the software ***snipar*** (Young et al., Nat Genet, 2022; Guan et al., Nat Genet, 2025), we simulated 20 generations of **assortative mating** before sampling trios, and again found our method to be robust to population stratification and assortative mating.

   - To evaluate **selection bias**, we showed that estimates of **direct effects are robust to non-random selection related to parental traits**, and that only asymmetric selection bias (e.g., traits affecting maternal choice for study participation) introduce bias in inference on asymmetric indirect effects. We also provide theoretical derivations to support these findings.

The detailed point-by-point response is below. The original reviewer's comments are in **blue**, and the responses below are in **black**. Major changes in the main manuscript are highlighted in yellow.

Reviewers' Comments:

Reviewer #1 (Remarks to the Author):

The authors extend prior methods for evaluating the association of polygenic risk scores (PRS) with binary disease status when sampling diseased cases and their parents. The strengths of this paper are the innovative statistical methods that address scientific questions about direct and indirect effects of PRS on a child's risk of disease. The benefits of the case-parent design, in terms of controlling for population stratification, are well known. The insights this paper provides, and the appropriate methods to do so, are the need to appropriately scale estimates of genetic effects to avoid bias, the ability to distinguish between direct and indirect effects of parental PRS on child's disease risk; the ability to evaluate whether the maternal and paternal indirect effects are the same; and the ability to estimate the interaction of direct genetic effects with environmental risk factors. The simulations demonstrate that the statistical methods control the false-positive rate and are unbiased, and perform as expected. The applications to the autism spectrum data (ASD) and the orofacial cleft data (OFC) nicely demonstrate how the methods can be used, and in particular provide some novel findings (e.g., maternal mediated indirect effects on risk of ASD in children; SNP x smoking interaction on OFC). The discussion is well-balanced in terms of highlighting the novel contributions of both statistical methods and results from the ASD and OFC analyses, as well as limitations of the methods. Overall, this paper is well written with appropriate tables and figures. I have a few relatively minor questions.

We would like to thank the reviewer for commenting on the strengths of the papers, including the distinct novel aspects of the methodologies.

1. Because logistic regression is commonly used for binary disease status, I think it would help the reader if you could explain why you use log-linear regression instead of logistic regression.
   Thank you for raising this important point. We chose the log-linear model because it yields closed-form estimators and allows for the direct estimation of relative risk parameters, which are more interpretable in many epidemiological contexts. Although logistic regression is more commonly used—primarily because it constrains predicted probabilities between 0 and 1—it estimates odds ratios, which may be less intuitive. For rare diseases such as the developmental conditions studied here, the relative risk and odds ratio tend to converge, making the two models nearly equivalent in practice. While logistic regression is standard in case-control designs, prior studies (Schaid, Genet Epi, 1999; Schmidt and Schaid, AJE, 1999) have shown that the log-linear model is more suitable for case-parent and

case-only designs, and rare disease assumptions are commonly invoked for comparing estimates with those from the logistic model. We have now clarified this in the **Material and Methods section (Page 5, line 139 and Page 6, line 140-149)**. We have also explained this in the **Supplementary Materials Page 2, Section 1.1.**

2. I think the justification of the assumption of fine-scale homogenous families needs more clarity. The authors state that their methods can account for assortative mating, but the assumption of random mating within the fine-scale homogenous populations seems unrealistic, as an attempt to circumvent the association between parents due to assortative mating. More clarity on this justification and why it accounts for assortative mating in real populations is needed. This is also relevant to the gene-environment independence assumption.

   We appreciate the reviewer's insightful comment. We believe that the introduction of family-specific parameters (μ_i, σ_i) to characterize the distribution of polygenic scores (PGS) without making any further distributional assumptions on these parameters makes our method highly robust to both population stratification and assortative mating (AM). In our framework, the notion of a "homogeneous family" is arbitrarily fine-grained and can be defined by a combination of population structure (e.g., ancestry, geography) and assortative mating-related characteristics (e.g., education). In fact, we point out that classical models for assortative mating also assume random mating conditional on trait characteristics (Falconer and Mackay, Introduction to Quantitative Genetics, 1996), thus have a lot of resemblance with our model. We use the concept of "homogeneous families" as a way to visualize the sampling scheme; however, the method does not require the existence of discrete population strata. In essence, each family can be considered its own stratum, ensuring general applicability.

   Importantly, we do not make any assumptions about the distribution of environmental exposures across families. From a practical standpoint, our modeling framework allows for strong population-level correlations between parental PGS (due to AM) and between PGS and environmental factors (due to stratification), through manipulation of the distribution of (μ_i, σ_i) across families. In our simulations shown in Figure 2a, the population-level correlation of PGS between parents across 1000 simulations was 0.69, and the population-level correlation between PGS and the environmental exposure E was 0.2 in Figure 2b for the GxE interactions —both of which are higher than typically observed in empirical data. In the UK Biobank simulations shown in Figure 3, the population-level correlation of education PGS between parents was 0.14. Under classical model for assortative mating, one also expects that the population level correlation

between PGS values of children and parents will be 0.5(1+r) where r is the correlation in PGS values of the parents. In fact, in our simulation data, the population-level parent-children correlation in UKB was 0.6, very close to the matching expectation.

In the revised version, we have now conducted an additional set of simulation using the software ***snipar*** (Young et al., Nat Genet, 2022; Guan et al., Nat Genet, 2025), to simulate 20 generations of AM with a phenotypic correlation of 0.5 between parents. The correlation of PGS between parents in this simulation was 0.3. These new results demonstrate that our method continues to yield unbiased estimates of both direct and indirect genetic effects even under strong and long-term AM scenarios (**Material and Methods, Page 11, line 298-303 and Page 12, line 304-305; Results, Page 15, line 418-422, Extended Figure 5**).

3. On line 18 of page 8, can you give an intuitive explanation of why the estimate of variance has '2" in the denominator? The rest of the equation makes sense, based on sample variance estimate.
   Because the parental difference variance is double the family variance. We define the difference as $X_i = PGS_{iM} - PGS_{iF}$, and we can show that it follows the distribution of $X_i \sim N(\delta_{MF}\sigma_i^2, 2\sigma_i^2)$. An unbiased estimator for $\hat{\sigma}_i^2$ will be half of the sample variance estimate of X_i. We have shown the complete derivation in **Supplementary Materials Section 1.2.1 and 1.2.2.**

4. On page 11, lines 21-21, state that PGS are standardized by ancestry-specific standard errors based on 1000 genome reference. How does this approach to standardization, which relies on self-reported ancestry, compare with the continuous PC projection approach available in the pgsc-calc (PGS Catalog)? Does your approach account for when parents are of mixed ancestry?
   In the revision, we have standardized the polygenic scores (PGS) using a principal component (PC) projection approach based on the 1000 Genomes Project and Human Genome Diversity Project (1000G + HGDP) reference panels. This revisualization procedure helps to partially remove the components of PGS that are attributable to population stratification. The detailed steps for this standardization procedure are provided in **Supplementary Materials Section 3.1.1.**

   We chose not to apply PC-adjustment to the *standard errors* of the PGS, since such an adjustment would transform the PGS into a non-linear function of genotypes. While Mendel's laws allow straightforward derivation of expected distributions for linear genotype-based scores in family-based designs, the same

is not true for non-linear constructs. Therefore, after performing PC-based mean standardization, we normalized the PGS by dividing them by ancestry group–specific standard errors. This approach enables valid comparisons of effect sizes across self-reported ancestry groups. For the SPARK consortium ASD study, the ancestry was inferred based on genetic reference panels; for the GENEVA OFCs study, the ancestry was based on self-reported ancestry, however, note that these case–parent trios were recruited largely through surgical treatment centers by multiple investigators from Europe (Norway), the United States (Iowa, Maryland, Pennsylvania, and Utah) and Asia (People's Republic of China, Taiwan, South Korea, Singapore, and the Philippines), so the self-reported ancestry (European and Asian) is mostly consistent with genetically-inferred ancestry due to the separation of geographical locations through the data collection process.

However, we acknowledge that this method is suboptimal in the presence of mixed-ancestry families, where parents differ in genetic ancestry—as is common in the SPARK dataset. In the revision, we now present a novel analysis that includes *both homogeneous and mixed families* in a unified framework without grouping them by ancestry. Instead, we incorporate an interaction term between PGS and the genetic distance of SPARK individuals from the training population of European ancestry. This allows for direct modeling of ancestry-modified genetic effects. **See Figure 5, Results, Page 16, line 437-447, and Table S5 for more details.**

This approach not only demonstrates the flexibility of our method in testing for PGS-by-context interactions, but also provides compelling evidence that the predictive performance of the existing autism PGS diminishes linearly (on the log-risk scale) as a function of genetic distance from the training population. While a recent study (Ding et al., *Nature*, 2023) observed a similar pattern in population-based analyses, our study is, to our knowledge, the first to replicate this effect using a purely family-based design.

5. Please explain the purpose of "Extended Figures". Are these to be in the supplement? If in the main article, there seem to be too many figures.

   The Extended Data Figures are intended to provide additional results for the simulation study (including 95% coverage probability, power evaluation, and additional sensitivity simulation studies) and data applications (qq plots, maps showing PGS and its geographical clusters) to demonstrate the robustness of our method, and supporting results that complement the main findings but are not essential for the primary narrative. These figures follow the format used by the Nature Genetics journal, where Extended Data Figures appear at the end of the main article (not in the Supplementary Information) and are numbered separately

(e.g., Extended Data Fig. 1, 2, etc.). We included 7 Extended Data Figures, which is within the allowed limit of 10. We understand the concern about the number of figures and have carefully structured the manuscript such that only the most critical visualizations appear as main figures. If preferred, we are happy to adjust this division or move some Extended Data Figures to the Supplementary Materials as appropriate.

Reviewer #1 (Remarks on code availability): I briefly reviewed the code, vignette, and help files. I installed the R package and ran the simulation in the vignette. All seemed to work fine.

Reviewer #2 and #3 (Remarks to the Author):

General Comments

The reviewers made some general comments regarding the novelty of the methods and their potential impact in the future. Below we group these comments together.

"There has been a lot of interest in family-based samples for estimating indirect effects. For example, Kong et al. (2018), used directly and indirectly transmitted polygenic scores to investigate indirect effects in a range of traits in deCODE. That was a bigger sample, and covered more traits. For Autism, Weiner et al. (2017) used polygenic transmission disequilibrium tests to investigate the transmission of various traits in 6,454 families. These are both referenced in the paper. The key contribution claimed for this study is that the previously cited papers did not present their results on an easily interpretable risk scale. This is a fairly standard rescaling of coefficients and doesn't represent a particularly major advance. E.g. see Tan et al. (2024) or Nivard et al (2024). Tan et al. 2024. 'Family-GWAS Reveals Effects of Environment and Mating on Genetic Associations'. Nivard et al 2024. 'More than Nature and Nurture, Indirect Genetic Effects on Children's Academic Achievement Are Consequences of Dynastic Social Processes'. Nature Human Behaviour 8(4):771–78. doi: 10.1038/s41562-023-01796-2."

The reviewers suggest that scaling of polygenic scores (PGS) for effect-size estimation is not a significant issue and has been addressed previously. However, after careful examination of the cited manuscripts, we found no discussion of the scaling problem in

the context of binary traits under ascertained study designs. Our work demonstrates that a rigorous theoretical framework—grounded in an underlying risk model—is essential to derive the correct scaling strategy. Without this, effect-size estimates from family-based studies cannot be meaningfully compared to those from population-based studies, potentially leading to widespread confusion in the literature regarding discrepancies in PGS effect sizes across study designs. We have now provided better intuition why transmission-based statistics need to be scaled by within-family variance parameters (see our responses below to your specific comments).

In addition, the reviewers appear to have overlooked another key contribution of our method: the development of a transmission-based approach for characterizing polygenic gene-environment interactions. To our knowledge, this is the first method of its kind. In our revision, we highlight its novel application to examine PGS-by-context interactions along the genetic ancestry continuum using SPARK autism data, demonstrating its practical utility.

"The results are not convincing, because the authors' central claim, that the indirect effects can be estimated from the polygenic scores is not demonstrated by their simulations or derivations. The key challenge is differentiating between effects due to assortative mating and population structure versus indirect effects. The authors essentially assume this problem away in their simulations without justifying why. They also do not address selection and collider bias. As a result, their simulations and empirical examples are not convincing."

In addition to deriving transmission-based estimates of the direct effect of PGS and PGS-by-environment interactions, another novel methodological contribution of our manuscript is the observation that differences in PGS values between parents of affected children can provide information on *indirect genetic effects*. Through theoretical derivations and extensive simulations—including new ones introduced in the revision—we demonstrate that the **difference in indirect effects between the two parents** can be estimated in a robust manner, even in the presence of population stratification and assortative mating. *Crucially, we emphasize that our framework estimates the* difference *in indirect effects by*

*parental sex, not the individual parental effects, which are more susceptible to confounding from population structur*e and assortative mating. Based on your recommendation, we have now further validated our results using simulations done based on the *snipar* package. Further, Dr. Alexandar Young, the author of the software *snipar* and multiple key papers in the field, also confirmed in personal communication that assortative mating is not expected to lead to differences in indirect effects between parents. We do acknowledge, however, that even such difference-based parameters may be sensitive to asymmetric selection of families based on maternal versus paternal choices, and we have conducted additional simulation studies and theoretical analyses to assess the impact of this potential bias.

We hope that the above has clarified the distinct methodological contributions of the paper.

Next, we address the specific comments.

Major comments

1. It would be worth updating the simulation to simulate the transmission of individual genotypes rather than the continuous scores. This simplification of the simulation means it omits the key point of randomized transmission of variants from parents to offspring.
   We would like to thank the reviewer for raising this up and would like to clarify that our original simulation studies comprised two components. The second simulation utilized individual-level genotype data from the UK Biobank, where we simulated pseudo-offspring individual genotypes. In addition, we have now conducted new simulations using the *snipar* software to model 20 generations of assortative mating with parental phenotype correlation 0.5. The results from these simulations have been incorporated into the revised manuscript (**see Material and Methods, Page 11, line 298-303 and Page 12, line 304-305; Results, Page 15, line 418-422, Extended Figure 5**). Overall, our findings demonstrate that the estimates produced by our method remain unbiased and robust across a range of population stratification and assortative mating scenarios.

2. The current paper doesn’t address selection or collider bias - this is another explanation for the results and can cause bias, even in samples of related individuals.
   Thank you for raising this important point, which we had previously overlooked. In the revision, we conducted additional simulation studies and theoretical investigations to examine the impact of selection/participation bias due to non-random sampling of families. In trio-based studies, it is plausible that certain parental characteristics—and therefore their genetic makeup—may influence a family’s likelihood of participating in research. We considered a selection

mechanism in which the probability of sampling a family depends on the parents' PGS values, but is independent of the child's PGS, conditional on those of the parents.

Under this model, we theoretically show that transmission-based estimates of the direct effect of PGS and PGS-by-environment interactions remain unbiased **(Supplementary Materials Section 1.3)**. However, if maternal and paternal PGS values differentially influence the likelihood of family participation, this *asymmetric selection* can bias inference on the **difference in indirect parental effects**—a key quantity we aimed to estimate. In such scenarios, observed differences in parental PGS may reflect true differences in indirect effects or differential selection into the study, or both. Our simulation results confirm these theoretical findings. We simulated 8 different scenarios when we assumed parents' PGSs can influence the family participation into the study, as shown in **Supplementary Materials Section 2.4**. We now explicitly acknowledge this potential source of bias and interpret the observed indirect effects—particularly those involving alternative trait PGS on autism risk—with appropriate caution in light of these caveats.

We refer the reviewers to **Supplementary Materials Sections 1.3, 2.4, and Supplementary Materials figures 1-3** for further details.

3. It would be useful to prove how it is possible to differentiate indirect effects due to the effects of parental phenotype and the effects of an environmental confounder or assortative mating. The following statement:
   "We conceptualize a sampling mechanism where for each family sampled, we assume it belongs to a unique subpopulation of "homogeneous" families with distinct genetic ancestry and trait characteristics influencing assortative mating. The number of these subpopulations can be arbitrarily large, making each subpopulation at an extremely fine level. It is assumed random mating occurs within these homogeneous fine-level subpopulations, implying the PGS correlation between partners is 0 within these subpopulations, but not necessarily across them."
   This approach is not explained in sufficient detail in the rest of the paper. The UK Biobank example does not adequately assess the robustness of this method to bias from assortative mating or population stratification. This should be assessed via simulations that induce assortment/confounding via structure into DGP.

   We believe that the introduction of family-specific parameters (μ_i, σ_i) to characterize the distribution of polygenic scores (PGS) without making any further distributional assumptions on these parameters makes our method highly robust to both population stratification and assortative mating (AM). In our framework, the notion of a "homogeneous family" is arbitrarily fine-grained and can be defined by a combination of population structure (e.g., ancestry, geography) and assortative mating-related characteristics (e.g., phenotype, education). We use the concept of "homogeneous families" as a way to visualize the sampling scheme; however, the method does not require the existence of discrete population strata. In essence,

each family can be considered its own stratum, ensuring general applicability. In fact, we point out that classical models for assortative mating also assume random mating conditional on trait characteristics (Falconer and Mackay, Introduction to Quantitative Genetics, 1996), thus have a lot of resemblance with our proposed model.

As noted above, our framework does not permit robust estimation of the *individual* maternal or paternal indirect effects without imposing strong assumptions on the distribution of the family-specific parameter μ_i across families. However, when estimating the *difference* in indirect effects between the two parents, these family-specific parameters cancel out. As a result, we argue that the difference parameter can be estimated in a more robust manner, even in the presence of unobserved heterogeneity across families.

We also do not make any assumptions about the distribution of environmental exposures across families. From a practical standpoint, our modeling framework allows for strong population-level correlations between parental PGS (due to AM) and between PGS and environmental factors (due to stratification), through manipulation of the distribution of (μ_i, σ_i) across families. For instance, in our simulations shown in **Figure 2a**, the population-level correlation of PGS between parents across 1000 simulations was 0.69, and the population-level correlation between PGS and the environmental exposure E was 0.2 in **Figure 2b** for the GxE interactions —both of which are higher than typically observed in empirical data. In the UK Biobank simulations shown in **Figure 3**, the population-level correlation of PGS between parents was 0.14. Under classical models for assortative mating, one also expects that the population level correlation between PGS values of children and parents will be 0.5(1+r) where r is the correlation in PGS values of the parents. In fact, in our simulation data, the population-level parent-children correlation in UKB was 0.6, very closely matching the expectation.

Nevertheless, based on your recommendation, in the revised version, we have now conducted a third set of simulation using the software ***snipar*** (Young et al., Nat Genet, 2022; Guan et al., Nat Genet, 2025), to simulate 20 generations of AM with a phenotypic correlation of 0.5 between parents. The correlation of PGS between parents in this simulation was 0.3, and the population-level parent-children correlation was 0.6. These new results demonstrate that our method continues to yield unbiased estimates of both direct effects and asymmetric indirect effects even under strong and long-term AM scenarios (**Material and Methods, Page 11, line 298-303 and Page 12, line 304-305; Results, Page 15, line 418-422, Extended Figure 5**).

4. Power - it would be worth running simulations to assess the relative power of these approaches as a function of R^2 of the trait of interest, and sample size.

   While R^2 is widely used to assess predictive performance for continuous outcomes in linear regression, for binary disease traits, the log odds ratio (log OR) or log relative risk (log RR), in sd unit scale, is generally sufficient to reflect the predictive strength of a PGS. In fact, measures such as AUC, which are commonly used to show discriminatory performance of model for binary conditions, is directly related to such relative-risk parameters (Pharaoh et al., Nat Genet, 2002). Unlike parameters such as relative-risks, R^2 for binary trait can be sensitive to disease prevalence and does not directly translate to measures of discriminatory performance of models for binary conditions.

   We originally included a power analysis based on log OR and sample size (**Extended Figure 2**). This analysis shows that our method achieves power comparable to that of the pTDT (Weiner et al., Nat Genet, 2017), a well-established transmission-based approach for testing PGS in case–parent trio designs, across a range of simulation scenarios.

5. ASD GWAS samples are small. How well did it predict ASD in the sample?

   We have expanded our ASD study sample from 1,517 to 18,383 trios from the SPARK consortium. Initially, our analysis was limited to a subset of trios with detailed information on maternal risk factors. In the revised analysis, we leveraged the full set of available trios for purely genetic analyses, while restricting PGS-by-environment (PGS×E) interaction analyses to 1,748 trios with corresponding exposure data.

   This substantial increase in sample size enabled a more robust characterization of the *direct effects* of established ASD polygenic scores (PGS) across ancestry groups and along the genetic ancestry continuum using transmission-based statistics. Consistent with prior findings in population-based studies (e.g., Ding et al., *Nature*, 2023), we observed that the predictive performance of ASD PGS declines continuously with increasing genetic distance between training and target samples. To our knowledge, this is the first family-based analysis to demonstrate this pattern, indicating that it is not merely an artifact of population stratification.

   The larger sample also allowed us to detect highly significant differences in PGS values for several traits between mothers and fathers of children with ASD. These findings support existing hypotheses—such as the causal effect of maternal obesity on ASD risk—and point towards potential for indirect effects of PGS for several autism related traits, but not autism itself.

   As shown in **Figure 4a**, the ASD PGS demonstrates highly significant direct effect on ASD risk in several populations except the African and East Asian ancestry populations. Among individuals of European ancestry, we estimate that each sd increase in a child's ASD PGS is associated with a 28% increase in risk (RR = 1.28, 95% CI = [1.25,1.31]). Using the formula from Pharoh et al., Nat Genet, 2002, this

corresponds to an estimated AUC of 0.6, reflecting the modest discriminatory power of current ASD PGS for predicting autism risk.

6. Have the authors ruled out gene-environment correlation when conducting the GxE interaction tests?

As noted earlier, we assess gene-environment interaction using a **transmission-based statistic**. Specifically, we show that the appropriately scaled covariance between the transmission disequilibrium statistic and environmental covariates across families yields an estimate of the multiplicative gene-environment interaction under our proposed model. While gene-environment correlation may exist at the population level—due to factors such as population stratification or assortative mating—the transmission of genotypes from parents to offspring should, in expectation, be independent of family-level environmental exposures. Therefore, under the null hypothesis of no interaction, the covariance between transmission and environmental factors should be zero. A significant departure from this "null" covariance provides evidence of multiplicative interaction. Importantly, this approach is **robust to gene-environment correlation** that arises from population structure, since it relies solely on within-family transmission. In the revision, we have now provided the above intuition following the formula for direct effect estimation (**Page 8, line 215-221 and Page 9, line 222-225**).

However, we acknowledge that PGS and certain environmental exposures may exhibit direct correlation not attributable to population stratification or assortative mating. If the environmental variable corresponds to **a trait of the child**, this can violate the key assumption that $\Pr(PGS_C|PGS_M, PGS_F, E) = \Pr(PGS_C \mid PGS_M, PGS_F)$ leading to biased interaction estimates. In contrast, if the environmental variable pertains to **parental traits**, the assumption remains valid, and the interaction analysis yields unbiased inference.

In the revision, we have clarified this important distinction and emphasized the limitations of using child-specific exposures in gene-environment interaction analysis when PGS and the exposure may be directly related in both Methods and Discussion. In our real data applications, the environmental factors primarily consist of **maternal lifestyle characteristics** or **child's sex**, which do not violate the core assumption, and thus we expect our results to be robust.

Minor Comments

1. Could you confirm whether there was any sample overlap between the GWAS used to generate the scores and the samples used in your analysis?

For the ASD data analysis, there is no known sample overlap between the GWAS used to generate the polygenic scores and the SPARK samples included in our analysis. The GWAS in question was based on a meta-analysis from the iPSYCH project (a Danish population-based case-control sample) and the Psychiatric Genomics Consortium (PGC), as reported in Grove et al., *Nature Genetics* 2019. The PGC analysis included five cohorts: the Geschwind Autism Center of

Excellence, the Autism Genome Project, the Autism Genetic Resource Exchange, the NIMH Repository–Montreal/Boston Collection, and the Simons Simplex Collection (SSC).

We confirm that our analysis is based solely on individuals from the SPARK study and that SPARK study did not contribute to the Grove et al. 2019 GWAS. Note, it is possible that participants enrolled in more than 1 study, e.g. SPARK and SSC or SPARK and Autism Genome Project. However, given the overwhelming number of PGC GWAS samples contributed by iPSYCH and likely relatively few US participants that were enrolled in more than 1 US study and unlikely they were also living in Denmark/iPSYCH, we would expect an extremely low number of our SPARK samples to have contributed to the discovery GWAS.

For the OFCs analysis, due to the restricted data resources available in previous GWAS efforts and the rarity of this developmental disorder, the PGS was constructed using multiple previous GWAS (Yu et al., Human Molecular Genetics, 2022). 10 SNPs were identified based on a meta-analysis using both GENEVA and POFC studies; 3 SNPs were based partially on GENEVA and Bonn GWAS studies. Nonetheless, these SNPs underwent subsequent validation using separate and independent data sources from the GENEVA study used in our analysis. The PGS was tested and reported in the PGS Catalog using the BioVU repository, independently from the GENEVA study. We explained this in the **Supplementary Materials Section 3.2.1**.

2. page 3 line 22 "randomly sampled families and are not" - could you confirm that these studies were randomly sampled families?
   Yes, we can confirm that most recent studies have developed methods under the assumption of *random sampling of families* and do not account for complex ascertainment. These methods are typically designed for continuous traits and often rely on random effects models to account for family structure. However, under non-random sampling schemes, the likelihood for such models can become quite intractable.

   In contrast, a key advantage of our approach—based on a log-linear disease risk model—is that, under the assumption of normally distributed PGS, we can derive a **closed-form likelihood** even in the presence of family-based ascertainment, as shown in our theoretical derivation. This makes our framework particularly well-suited—or even "canonical"—for the analysis of ascertained case-parent trio data and ascertained families more broadly.

3. Page 4 line 24 "We obtain transmission-based estimates of effect sizes for PGS underlying these two traits and show that they are comparable to corresponding effect sizes reported in prior studies, primarily conducted based on unrelated cases and controls". Could present a direct comparison of your estimates with those from prior studies?

We included the comparisons with previous population-based case-control studies in the main text, **Page 16 line 426-429** for the ASD study; and **Table S13** for OFCs. Note that the OFCs estimates were reported in PGScatalog using the BioVU study (46 cases, 72,496 controls) as AUC=0.66 for European CL/P, we converted this to log RR as around 0.6. Our estimated log RR for European CL/P is 0.715.

4. Page 4 line 29 “For both ASD and OFCs, we examine PGSxE interactions for several known maternal risk factors”. How did the results compare to estimates from other studies? To what extent was the variance reduced.
   The maternal risk factors included in our analysis are known to be associated with ASD and OFCs risk at the main effect level. To the best of our knowledge, there are no prior studies systematically investigating PGSxE interactions for these conditions.

5. Page 6 line 16 “Finally, we assume $EiC \perp (PGSiC, PGSiM, PGSiF)$, but allow the distribution $\boldsymbol{EiC}$ to remain unspecified. From a population perspective, this assumption can again be viewed as “gene-environment” independence within highly homogenous subpopulations” and “For the investigation of gene-environment interactions, we incorporate a binary ($E1$) and a continuous ($E2$) variable in the disease model. We allowed complex inter-relationships among baseline disease risk (αi), PGS mean (μi) and exposure means ($\gamma i1$ and $\gamma i2$) across families,” -How does the model account for the variance in E between families, which impacts GxE. Why do you assume gene-environment independence, is this assumption plausible? To what extent are you simply assuming confounding away in your models and simulations?
   We are not entirely sure we understand the reviewer’s concern regarding the impact of the variance of the environmental variable (E) on the gene-by-environment (G×E) interaction effect. If the comment pertains to the role of E’s variability in explaining phenotypic variance in *continuous traits*, that interpretation is not directly relevant to our current setting. Our goal is ***not*** to quantify the contribution of interaction to phenotypic variance, but rather to test whether the *effect* of a given PGS on disease risk is *modified* by the level of an environmental factor—i.e., whether there is a multiplicative interaction on the risk scale. This aligns with the standard definition of statistical interaction in epidemiologic and genetic studies.

   Regarding the assumption of gene-environment independence, we refer the reviewer to our response to *Major Comment 6*, where we discuss in detail the robustness of our transmission-based interaction statistic to gene-environment correlation due to factors such as population stratification.

6. Page 8 - line 22 - Why do you make this modification?
   In the presence of asymmetric indirect effects, the difference in PGS values between mother and father does not have mean zero. Therefore, when calculating the variance of this difference, it is necessary to **mean-center** the expression to

obtain an unbiased estimate. The details of the derivations are in **Supplementary Materials Section 1.2**.

7. Page 9 line 19 - "Known to be highly confounded with environmental factors" - did these studies prove which of pop strat, indirect effects and assortment confounds and explains these associations?
   Previous research such as Okbay et al., Nat Genet, 2022 have shown that the direct effects explain roughly half the PGS's magnitude of association with Educational attainment (EA) (after controlling for parental PGSs). This indicates the confounding came from both indirect effects and assortment. Another work Guan et al., Nat Genet, 2025 used family-based methods to show the contribution of indirect effects and AM and also showed there is residual population stratification in standard GWAS. Abdellaoui, et al., Nature Human Behaviour, 2019 has shown that EA PGS is highly confounded by geographical regions, after controlling for ancestry. This probably reflects migration driven by socioeconomic status (SES). The level of geographic clustering is correlated with genetic associations between complex traits and regional measures of SES. This evidence led us to choose EA-PGS in our simulation study to create realistic population stratification and assortment to comprehensively assess our method.

8. Page 9 line 21 - Why did you create your score based on PGS002012? Really the key parameter here is R^2, and the LD structure etc, is not terribly relevant.
   We agree that, for real data analysis, the key parameter influencing power is the R^2 between the PGS and the trait, rather than linkage disequilibrium (LD) structure itself. In our simulations of UK Biobank offspring data, we restricted the analysis to a set of independent SNPs to simplify the simulation process. Specifically, simulating genotypes at independent loci can be done using Mendel's law without accounting for haplotype structure. In contrast, simulating SNPs in LD would ideally require modeling underlying haplotypes and their transmission, which is considerably more complex. Therefore, we chose to use independent SNPs purely for **computational convenience**, without implying that LD is irrelevant for real data applications. We further explained this in the next comment. This can also be found in **Supplementary Materials Section 2.2**.

9. Page 9 - how did you simulate assortative mating? E.g. what functions and code?
   We simulated assortative mating using two sets of studies, one is the UK Biobank and the other is *snipar*. For the UK Biobank study, the assortative mating was created by using educational attainment (UKB Field ID: 6138) as one of the criteria (in addition to geographic proximity, UKB Field ID: 54, 129, and 130) to match male and females to create "pseudo" parents in UKB. It has been previously reported that EA is a common trait in assortative mating and the EA-PGS are heavily confounded by geographical regions. In the revision, following the reviewers' suggestion, we ran an additional simulation using the software *snipar* to simulate 20 generations of assortative mating. The programming codes are available in Github repository

https://github.com/ziqiaow/PGS-TRI-Analysis/tree/main/2_simulation/UKB
The new simulation codes which include *snipar* and selection bias are available in https://github.com/ziqiaow/PGS-TRI-Analysis/tree/main/6_revision1/simulation

10. Page 9 - Mendelian transmission - could you simulate the transmission of genotype rather than the transmission of the normally distributed continuous liability?
We apologize for not making this clearer in the original version, and we thank the reviewer for pointing it out. This is exactly what we did for the UK Biobank simulation (also see our response to *minor comment 8*). We have revised the sentence to clarify that we simulated genotype transmission (see **Page 11, lines 292-294**). We hope this revision improves the manuscript's clarity.

11. Page 10 line 19. "As a negative control, we used body mass index (BMI)" Why is BMI considered a negative control? Many of these conditions are associated with BMI, especially depression, SCZ (positively) and ED (negatively). Why not height?
We agree that height is also a reasonable choice for a negative control. However, we selected BMI-PGS as a negative control for *direct effects* on ASD risk, consistent with prior work, including the pTDT study by Weiner et al. (*Nat Genet*, 2017). Importantly, BMI has been the focus of persistent epidemiologic evidence linking **maternal BMI** to increased autism risk in offspring. In the current revision, using a much larger ASD trio dataset, we confirm that BMI-PGS shows **no evidence of a direct effect** on ASD risk in children—reinforcing its role as a negative control in this context.

However, we do find highly significant **evidence of a maternally mediated indirect effect** of BMI-PGS on ASD risk, with the direction of association aligning with findings from observational studies. Thus, BMI-PGS serves as a compelling example of a genetic factor that functions as a **negative control for direct effect**, but a **positive control for indirect (maternal) effect**—highlighting the utility of our analytic framework for disentangling these distinct pathways.

12. Page 10 - line 22 - "We standardized all of the PGSs by ancestry-specific standard errors obtained from the 1000 23 Genomes (1000G) Phase 3 Project.24" How did you standardize the PGS? By what SE? How does this allow for interpreting the underlying risk parameters on the standard risk scale?
For analyses stratified by ancestry, we standardize each PGS by its standard deviation **within each group**. As previously noted, relative risks associated with PGS (expressed in standardized units) directly relate to the **discriminatory performance** of the score for binary outcomes. Thus, scaling the association coefficients by the group-specific PGS standard deviation facilitates comparison of expected discriminatory power across those ancestry groups.

For the ASD analysis, we have now added a new analysis that does **not categorize families into discrete ancestry groups**, but instead analyzes all families in a **pooled framework**, allowing the **relative risk associated with the**

PGS to vary as a function of the **genetic distance** of each individual case from the training population used to derive the original PGS. In this pooled analysis, we examined interactions between genetic distance and PGS, using both group-specific standardized PGS (by standard deviations) and original unstandardized PGS. **See Figure 5, Results Page 16, line 437-447, and Table S5 for more details.** This approach not only demonstrates the flexibility of our method in testing for PGS-by-context interactions, but also provides compelling evidence that the predictive performance of the existing autism PGS diminishes linearly (on the log-risk scale) as a function of genetic distance from the training population. While a recent study (Ding et al., *Nature*, 2023) observed a similar pattern in population-based analyses, our study is, to our knowledge, the first to replicate this effect using a purely family-based design.

Additionally, in the revised analysis, we have **mean-standardized** the PGS using the widely adopted **PC projection approach**, leveraging the 1000 Genomes Project and Human Genome Diversity Project (HGDP) reference datasets. This step helps mitigate spurious variation in PGS due to population stratification. Please see **Supplementary Materials Section 3.1.1** for further methodological details.

13. Page 10 - line 29 - Random transmission of the ASD score should ensure independence of the ASD score and the OMICSPRED gene expression and metabolite PRSs. If you do find a direct association here, then this suggests that your data may suffer from selection and collider bias.

We may have misunderstood the reviewer's concern. Our analysis tests whether **omics genetic scores or biomarker-PGSs** (not the ASD PGS) have **direct or indirect effects** on ASD risk. Under the null hypothesis of no direct effect, we expect Mendelian transmission of these PGSs from parents to children; deviations from this pattern indicate direct effects. Similarly, we can test for the indirect effect of parental PGS of biomarkers on children's risk.

We acknowledge that ideally, biomarker-PGS should be trained on data from relevant **developmental stages for investigation of direct effects**. However, genetic predictors of many biomarkers are known to be stable across life stages (see Gaunt et al., Genome Biology, 2016), making it likely that OMICPRED PGS trained in adults will retain some predictive value in earlier life. If these PGS had no power for predicting the biomarker value in relevant developmental stage, we will only expect null results, and non-null results will be suspicious.

In our **OFC analysis**, we found a direct effect for gene expression PGS at **TRAF3IP3**, a locus previously implicated in OFC GWAS. In the expanded **ASD sample**, we identified direct effects for two gene-expression PGS, including **CADM2**, a gene with known roles in psychiatric traits and neuronal signaling.

Given the limitations of using OMICPRED PGS to study direct effects on developmental outcomes, we have not emphasized these results as major findings of the paper. Instead, we present them as a **proof of concept** to illustrate the potential application of our method for large-scale, omics-derived PGS analysis. Looking ahead, as PGS for omics traits become available across diverse contexts—such as different life stages, tissues, and cell types—our framework can be used to investigate **direct and indirect effects** in family-based studies.

14. Page 13 - line 21 - "low power and perhaps lack of 21 transportability of the PGS to non-EUR populations (Table S5)." Did you find any evidence that the effect of the PGS on the trait in question (if measured) in these populations differed?
    With the expanded ASD trio dataset, we were able to clearly demonstrate that the predictive effect of ASD PGS on ASD risk **declines with increasing genetic distance** between the test and training populations. Prior multi-ancestry studies—mostly using unrelated individuals—have consistently shown that PGS trained in European populations perform poorly in non-European groups, particularly those of African ancestry. Notably, Ding et al., *Nature*, 2023, quantified this degradation as a function of genetic distance. To our knowledge, ours is the **first family-based study** to replicate this key finding, confirming that the decline in PGS performance with genetic distance is **not an artifact of population stratification**.

    Due to the lack of phenotype data on non-ASD traits in our sample, we were unable to evaluate cross-ancestry performance for PGS for the prediction of non-ASD traits.

15. Page 13 line 29 - How can you differentiate the maternal effects from assortment on that trait in the parental generation?
    As previously explained, we do not estimate maternal effects directly; rather, we focus on the **difference between maternal and paternal indirect effects**—a parameter that is more robust to **assortative mating**. Since maternal and paternal effects are not necessarily symmetric, this difference is both statistically informative and biologically meaningful. Importantly, it allows for improved control of confounding from assortative mating while still retaining **power to detect indirect effects**, assuming those effects differ between parents.

16. Somewhere it'd be good to include a directed acyclic graph encoding the assumptions you're making, and potentially comparing them to other approaches such as sibling regression.
    We included **Figure 1** to illustrate our PGS-TRI model framework for case-parent trio family study designs, which works similar to a directed acyclic graph. Specifically, **Figure 1a** shows the needed data input for the analysis (case-parent trios genotype data and environmental exposures if GxE analysis is needed).

Figure 1b shows the model assumptions we made in our approach (population-level disease model and family PGS distribution), and the model parameters we are able to estimate in PGS-TRI.

17. Page 17 - line 10 - the intuition for why this rescaling is necessary is not clearly explained in the paper. I agree rescaling is necessary, but why by the within family variance in the PGS?

Thank you for pointing out that the intuition behind the scaling was not clearly explained. While the mathematical derivation shows that the transmission statistic must be scaled by a measure of within-family variance, the underlying rationale is as follows:

In all ascertained study designs—including case-control, sibling case-control, and case-parent trio studies—we are essentially comparing PGS values of cases against appropriate "controls" or "pseudo-controls" to assess the strength of association. The *mean difference* in PRS values between cases and controls reflects not only the effect size (β), but also the *variance* of the PGS in the underlying population from which cases and controls are sampled. Therefore, even if the association strength of a given PGS is identical, the observed contrast between unrelated cases and controls will generally be larger than that between related individuals (e.g., in family-based designs), simply because the between-individual variance is greater.

For instance, in settings with population stratification that does not confound the disease association (see **Figure 2a,c** upper panels), the effect size may be the same within and between families. However, the variance of the PGS differs across those contexts, leading to different magnitudes of contrast. Thus, to obtain a comparable and unbiased estimate of the underlying association parameter, the case-control contrasts must be **appropriately standardized** using variance terms relevant to the study design.

We have now added a brief version of this intuition to the main text (**Page 8, line 206-214**).

18. Page 18 - line 5 - but surely the maternal BMI score is far better powered than the metabolite scores? Larger GWAS, but perhaps it explains less of the variance in BMI than the metabolites? This would be a very nice point to make (more proximal measures like metabolites can provide more accurate results with smaller samples).

We confirm that in the most updated analysis with much larger sample sizes of case-parent trios, we observed the maternal BMI indirect effect on ASD risk, while the metabolite genetic scores/PGS become less significant.

19. Page 19 - line 11 - “not well predicted” - MR analyses would test this association, either using summary data or IPD, can you provide some similar guidance here, is it possible to test whether the PGS sufficiently predict your exposure?

    Here, we were referring to the fact that the original **OMICSPRED models were trained on adult samples**, whereas for assessing **direct effects** in our analysis, the ideal biomarker PGS would be derived from **child-specific data**. This mismatch between the *training* (adults) and *testing* (children) populations may reduce the accuracy of biomarker prediction in children and thus lower the power to detect associations.

    Unfortunately, we currently lack access to transcriptomic and metabolomic data in children to directly evaluate how well adult-trained PGS predict the same traits in early life. In the future, resources like the **developmental GTEx** project (see https://gtexportal.org/home/aboutDGtex) may help fill this gap. Still, we expect some degree of **predictive transferability**, as genetically regulated components of biomarker traits often remain stable across life stages.

Response to referees

We are grateful that three of four reviewers are satisfied with the current version. We also thank for the fourth reviewer for several insightful comments. Following are our point-by-point responses. The original reviewer's comments are in **blue**, and the responses below are in **black**. Changes in the main manuscript are highlighted in yellow.

Reviewer #4 (Remarks to the Author):

The manuscript by Dr. Wang and colleagues present PGS-TRI, a method that leverages genetic and phenotypic data from trios (both parents and an child) to estimate direct, indirect, and gene-environment effects. The major benefit of trios is robustness to population stratification and assortative mating, when analyzing the child's phenotype conditional on the parental genotypes. This has been appreciated for many years and many statistical methods have been developed around this idea, which is a blueprint this manuscript follows.

We thank the reviewer for acknowledging the importance of family-based designs, and trio studies in particular, for genetic epidemiologic research. As we have noted and extensively referenced throughout the manuscript (see, in particular, **Page 3, Lines 61-67 in the Introduction and Pages 11-12, Lines 304-324 in the Discussion**), family-based studies have a long history, and numerous methodological frameworks have been developed for analyzing individual genetic variants and multi-allelic haplotypes.

Our contribution is distinct in that it addresses an emerging and rapidly evolving area: the analysis of polygenic risk scores (PGS) within family-based designs. Despite the extensive prior work on variant-level methods, there remain very limited methodological tools for PGS analysis in trios. In particular, no existing approach can jointly leverage all available components of family-based data to estimate polygenic direct effects, gene–environment interactions, and indirect (parental) genetic effects in a unified framework. Our work fills this methodological gap and provides a comprehensive strategy for extracting the full inferential potential of trio data for polygenic score analyses.

Unfortunately, I found the manuscript hard to follow and the analyses scattered across many datasets, datatypes, and hypotheses. I do not think this manuscript is a good candidate for publication in Nature Genetics. In my view, the major weaknesses are: 1) limited applicability of the method and 2) limited interesting findings when the method is applied to the largest datasets the method is applicable to. I highlight major issues below:

Limited applicability of the method:
While trio data is very powerful, the sample size has remained small. It may be true that more data is coming, but as it stands I do not think the method is of interest to the broad readership of Nature Genetics.

For the autism study, we analyzed data from **18,383 case–parent trios** with substantial ancestral diversity. This represents a very large sample size even by contemporary GWAS standards. Importantly, with this level of data, we have already been able to make

several notable observations, demonstrating that trio-based designs at this scale yield meaningful insights and clearly illustrate the scientific utility of such studies.

Limited interesting findings:
While gene-environment interactions are a primary interest of the method (it is in the title of the paper), the authors do not find convincing evidence (i.e. strong p-values) in real data for GxE for the two main disease traits they study, autism spectrum disorder and orofacial cleft. The authors claim that a major novelty of their method is the GxE component, but in their application of the method they do not find significant hits (see below). The authors do find evidence of direct genetic effects, but these do not seem novel. The authors find evidence for indirect genetic effects, although I am not convinced by them (see below). Other findings have been previously reported, for example the TRAF3IP3 association with OFCs (PMID: 33137956, Ref. 46 in manuscript). (Similar for CADM2). Taken together, the findings are of limited interest.

Studying gene–environment interactions is one—though not the only—primary objective of our paper. The contribution of the paper is the development of a comprehensive method for analysis of PGS in trio studies to characterize direct and indirect effects of PGS and PGS by E interactions. Our applications provide the first transmission-based estimate of effects for established PGS for ASD and OFC across diverse ancestry populations, identify effects of PGS for obesity and neurocognitive traits for ASD risk, and characterize the nature of PGS by context and PGS by environment interactions for two neurodevelopmental traits.

With respect to gene–environment interaction analyses specifically, our goal is not merely to identify statistically “significant” interaction terms, but to characterize the fundamental ways in which genetic and environmental factors jointly influence ASD and OFC risk. We agree that, after accounting for multiple testing, we do not observe strong evidence of non-multiplicative interactions. We did not highlight “significant” interactions and instead we noted in the Discussion that our results indicate that genetic and environmental factors generally act multiplicatively. This finding—although a “non-finding” in a narrow statistical sense—has important implications for public health. In the revision, we have now further presented power analysis for G by E study (**Supplemental Notes Section 2.5, Fig.6,7**), which indicates we can rule out substantial non-multiplicative effects. When genes and environment act multiplicatively on risk, environmental exposures tend to have their largest absolute-risk impact among individuals and families with the highest genetic susceptibility. This pattern has been demonstrated in adult-onset diseases such as breast cancer and cardiovascular disease, but has been far less explored for neurodevelopmental conditions. The contribution of our study lies in elucidating how polygenic and environmental factors combine, not in the pursuit of isolated statistically significant interaction parameters. In the revised manuscript (see **Page 14, Lines 381-389**), we have now highlighted these issues in a new paragraph in the discussion.

Lack of rigorous statistical control:

The authors report nominal significance in their main figures and the main description of the results largely excludes reporting p-values. This is not acceptable. Correction for multiple hypothesis testing is standard in the field. This has implications for the findings reported in the mauscript: In Table S7, for indirect genetic effects I see 5 tests that pass a Bonferonni threshold in the Cross-Population test, while the authors report 10 significant tests.

We acknowledge that we observed only borderline evidence of non-multiplicative interactions for a subset of environmental factors, and these signals do not withstand rigorous multiple-testing adjustment. As noted above, and as stated in the Discussion, our overall conclusion is that genetic and environmental factors generally act multiplicatively in influencing ASD and OFC risk.

With respect to indirect effects, our findings were considerably stronger. As the reviewer correctly noted, 5 of the 10 tests remained significant after Bonferroni adjustment and we have now clarified both in the text of Results section (**Page 8, Lines 217-219**) and **Figure 4 legend**. We, however, note that associations between PGS for non-ASD traits and ASD risk have been reported previously in population-based studies. Thus, our analyses should be interpreted not as a discovery exercise—where strict multiple-testing correction is required—but rather as a confirmatory evaluation using an orthogonal family-based design.

PGS x Context analysis:

I do not think this analysis tells us anything about the genetic architecture of autism. I could not find an equation corresponding to what the p-value < 1e-4 in Figure 4C. I do not understand the motivation for this analysis in this manuscript, as ancestry is not directly relevant for PGS-TRI. Further, the interaction itself is not interesting because many scenarios could lead to significant PGS x Context interactions. At a minimum, the authors should convice the reader that this is not due to scale effects. For example, consider the following R code that models additive effects on the liability scale, but leads to a significant PGS x Context term in a regression:

```
> N=1000
> PGS = rnorm(N)
> Context = rnorm(N)
> L = PGS + Context
> Disease = L < 3
> summary(lm(Disease ~ PGS*Context))
```

Can the authors rule out a model like this?

The ancestry dependence of PGS performance across diverse contexts—including gradients along genetic ancestry axes—has been a major focus of post-GWAS research (Martin et al., Nature Genetics, 2019; Duncan et al., Nature Communication, 2019; Ding

et al., Nature 2023). Notably, Ding et al. recently published an influential article in *Nature* demonstrating that PGS predictive performance declines continuously as a function of genetic distance between the training and test samples. This finding has important implications for the development, calibration, and application of PGS in diverse populations. However, the analysis of Ding et al. was based entirely on unrelated individuals and may still be susceptible to residual confounding from population stratification or assortative mating.

In contrast, our transmission-based framework PGS-TRI provides a design-robust assessment of PGS transferability. Using this approach, we show that **within-family** predictive effect of the ASD PGS decreases continuously with increasing genetic distance between the training and test samples, fully consistent with the pattern observed by Ding et al., but now demonstrated in a setting that is immune to population stratification and assortative mating biases. This substantially strengthens the evidence that the observed decay in PGS performance is not an artifact due to population stratification or assortative mating. For the results reported in Fig.5c, we used PGS-TRI to estimate PGS x E interaction, where Context is the E here. Detailed results are in Table S5.

For ASD—a binary trait—we focus on a canonical *relative-risk* (log-linear) scale, expressed in standard deviation units of the PGS. This is the standard scale used in the PGS literature, and the effect size on this scale maps directly to measures of classification performance such as AUC, which are of substantive clinical relevance (Pharaoh, Nature Genetics, 2002). Thus, from a predictive-performance perspective, our findings fully align with expectations regarding PGS transferability across genetic ancestries. We agree, however, that these interactions should not be interpreted mechanistically, and we have clarified this explicitly in the Discussion (**Page 13, Lines 350-351**).

The reviewer asks whether we could analyze disease risk on a linear scale. Our current model is formulated on the relative-risk (log-linear) scale because this leads to closed-form estimators and is the canonical parameterization for case–parent trio designs. We agree that developing flexible models that allow gene–environment and gene–context interactions to be parameterized on alternative scales is an important future direction. However, the linear scale suggested by the reviewer poses substantial challenges for binary traits, particularly when families are ascertained on disease status. Under such ascertainment, linear-scale effect estimates can be severely distorted, whereas relative-risk and odds-ratio parameters are far more robust. To obtain population-interpretable effects under a linear model, one would need to specify the disease prevalence in the underlying population—and these estimates can be highly sensitive to the assumed prevalence. Moreover, for our PGS-by-context analyses, the linear-scale formulation would require prevalence estimates across the genetic-ancestry continuum, which are not currently available. By contrast, our transmission-based relative-risk model does not require *any* assumptions about disease prevalence, making it well-suited to case-ascertained trio studies.

For these reasons, we believe there are clear advantages to modeling effects on the relative-risk scale in family-based analyses of binary traits.

Choice of traits analyzed:
I could not find a justification for the set of traits analyzed in this manuscript, which seem random. In addition, the TWAS analysis is not well-motivated.

We selected these traits primarily because prior epidemiologic and genetic studies have consistently linked them to autism risk, not to mention some of the notable works including the pTDT (Weiner et. al, Nat Genet 2017), and cross-trait LD score regression (Bulik-Sullivan et. al., Nat Genet 2015). We have cited the relevant literature in the revised manuscript (**Page 23, Lines 633-639).**

Regarding the TWAS analysis, we intended to illustrate the types of downstream integrative analyses that could be enabled by family-based PGS frameworks in future work. The key aspect of applying our framework in TWAS is to first generate genetic scores for each gene and then use PGS-TRI to assess the association between ASD/OFC risks and each genetically predicted gene. This application demonstrates how our method can be used to explore potential causal relationships or Mendelian randomization–type analyses within our framework, allowing molecular-level inference rather than limiting analyses to trait-specific PGS associations with diseases. As we already acknowledge in the Discussion (**Pages 14-15, Lines 407-411**), the current data are not ideally suited for investigating direct effects of PGS for molecular biomarkers on developmental traits, since the available omics summary-level data are primarily derived from adult rather than pediatric samples. For this reason, we did not emphasize or interpret any specific TWAS findings in the manuscript. Instead, we present the TWAS component as a proof-of-concept example, outlining potential extensions once larger and more appropriate datasets become available.

Selection bias results and differences in male female ascertainment (Supplementary section 2.4).
The authors show strong bias in the method for detecting indirect genetic effects when there is differential selection bias across the parents, which is sensible given their test statistic is testing for differences in PGS between the parents. However, the authors do not do any analyses testing the sensitivity of their Figure 4B results to this kind of bias or explain why we shouldn't worry about that kind of bias.

We did conduct a sensitivity analysis using external UK Biobank (UKB) data to assess whether the PGS for these traits exhibit any sex-specific shifts in the general population, and we further developed an PGS-TRI-centered approach using reference data to correct for potential allele frequency differences in the underlying population. Specifically, we assessed the UKB PC-standardized differences between females' and males' PGSs in unrelated European individuals who have been married. Marriage status derived using self-reported household status living with their husband, wife or partner (ID: 6141). N(total) = 250,066. N(female) = 128,827, N(male) = 121,239. While we observed small sex differences for a few traits, these patterns are far too modest to account for the magnitude or direction of the indirect effects observed among parents of autistic children

(**see Supplementary Table S20; Page 13, Lines 362-366**). We further used the PGS-TRI-centered approach and showed consistent IDE effect for most traits, with some even stronger effects in the European trios (see **Supplementary Table S7 (column I-K)**). These results reinforce that the parental patterns in our study are unlikely to be driven solely by baseline sex differences in PGS distributions.

Nonetheless, as noted in multiple places in the Discussion (**Page 13, Lines 354-366; Page 14, Lines 403-407**), we remain appropriately cautious in interpreting the indirect-effect estimates, given the potential for selection bias in case-ascertained family studies.

Less Major Issues:

- Line 156: "We assume within a family, PGS for the parents are independently distributed" Why is this assumption necessary? Does assortative mating lead to issues because of this assumption?

We refer to the reviewer for a detailed explanation of the issues in the text (**Pages 16-17, Lines 462-471 in Online Methods**). Also we showed the robustness of the proposed method against assortative mating under multiple simulation schemes, including using the UK Biobank data and *snipar* software (**Online Methods, Pages 21-22, Lines 587-610; Results, Pages 6-7, Lines 156-176, Extended Fig.4,5**).

- Line 205: How can you get accurate estimates of the within-family variance given that trios only contain 3 data points?

The denominator of the derived main effect estimator in Equation (4) actually requires estimating the average of within-family variances across all families. Thus, while estimating within-family variance for each family using only 3 data points is expected to be imprecise, the estimate of such quantities averaged across families can be quite precise when the number of families are large.

- Citation in the response to reviewers of a personal communication to Dr. Alexander Young is not convincing.

We understand this and that is why we did not cite it in the paper. We actually first conducted simulations using the software developed by Dr. Alexander Young et al., to show that our method is robust to assortative mating (**Online Methods, Page 22, Lines 603-610; Results, Page 7, Lines 173-176, Extended Fig.5**). The communication with Dr. Young occurred during the analysis, when we reached out to him for assistance with his software and engaged in a follow-up conversation.

- Line 450: "indicating polygenic risk and environmental factors generally act multiplicatively on the risk of ASD." Have the authors ruled out lack of statistical power as an explanation?

Great point. We have performed simulations assessing power with respect to multiplicative PGSxE interactions for both binary and continuous variables (**see Supplemental Notes Section 2.5, Fig.6,7**). As shown in the simulation, a relatively small trio size of 1,000 can induce 90% power for true log RR of 0.2 (RR = 1.2) for PGS x E interaction effect for a **continuous E**; and a trio size of 2,000 can induce 90% power for true log RR of 0.4 (RR = 1.5) for PGS x E interaction effect for a **binary E**. In our data applications for OFCs and ASD, both contain more than 1,700 complete trios in cross-population for the PGS x E interaction analyses. However, increasing the number of trios in future studies would allow stronger validation and improved characterization of PGSxE interactions for sure.

- Extended Figure 5B: inflated false positive rate in null simulations. On line 418, the authors claim that PGS-TRI remains unbiased in the presence of assortative mating, but this appears at odds with Extended Figure 5B, which shows bias in the presence of assortative mating.

We would like to clarify that in Extended Fig.5b, the type I error rate for the indirect effect for maternal and paternal PGS is shown based on logistic regression of case-control studies of unrelated individuals, where we assume parental genotypes are also available. This result demonstrates that under assortative mating, the individual estimates of each parent's PGS effect can yield false positives. However, in this paper, though we point out that our target is to infer parental asymmetry in indirect effects and this is more robust to the effect of population stratification and assortative mating. Indeed, our simulation shows that the difference in indirect effects of the two parents is not susceptible to the bias due to assortative mating and population stratification.
